# Neuroprotective Potential of Mesenchymal Stem Cell-Based Therapy in Acute Stages of TNBS-Induced Colitis in Guinea-Pigs

**DOI:** 10.1371/journal.pone.0139023

**Published:** 2015-09-23

**Authors:** Ainsley M. Robinson, Sarah Miller, Natalie Payne, Richard Boyd, Samy Sakkal, Kulmira Nurgali

**Affiliations:** 1 Centre for Chronic Diseases, College of Health and Biomedicine, Victoria University, Melbourne, Australia; 2 Department of Anatomy and Neuroscience, Monash University, Melbourne, Australia; 3 Australian Regenerative Medicine Institute, Monash University, Melbourne, Australia; National Institutes of Health, UNITED STATES

## Abstract

**Background & Aims:**

The therapeutic benefits of mesenchymal stem cells (MSCs), such as homing ability, multipotent differentiation capacity and secretion of soluble bioactive factors which exert neuroprotective, anti-inflammatory and immunomodulatory properties, have been attributed to attenuation of autoimmune, inflammatory and neurodegenerative disorders. In this study, we aimed to determine the earliest time point at which locally administered MSC-based therapies avert enteric neuronal loss and damage associated with intestinal inflammation in the guinea-pig model of colitis.

**Methods:**

At 3 hours after induction of colitis by 2,4,6-trinitrobenzene-sulfonate (TNBS), guinea-pigs received either human bone marrow-derived MSCs, conditioned medium (CM), or unconditioned medium by enema into the colon. Colon tissues were collected 6, 24 and 72 hours after administration of TNBS. Effects on body weight, gross morphological damage, immune cell infiltration and myenteric neurons were evaluated. RT-PCR, flow cytometry and antibody array kit were used to identify neurotrophic and neuroprotective factors released by MSCs.

**Results:**

MSC and CM treatments prevented body weight loss, reduced infiltration of leukocytes into the colon wall and the myenteric plexus, facilitated repair of damaged tissue and nerve fibers, averted myenteric neuronal loss, as well as changes in neuronal subpopulations. The neuroprotective effects of MSC and CM treatments were observed as early as 24 hours after induction of inflammation even though the inflammatory reaction at the level of the myenteric ganglia had not completely subsided. Substantial number of neurotrophic and neuroprotective factors released by MSCs was identified in their secretome.

**Conclusion:**

MSC-based therapies applied at the acute stages of TNBS-induced colitis start exerting their neuroprotective effects towards enteric neurons by 24 hours post treatment. The neuroprotective efficacy of MSC-based therapies can be exerted independently to their anti-inflammatory effects.

## Introduction

Chronic relapsing inflammation of the gastrointestinal (GI) tract is characteristic of inflammatory bowel disease (IBD) which includes two pathologies, Crohn’s disease and ulcerative colitis. The onset of IBD is concomitant with young adulthood and in its active stage presents through abdominal pain, diarrhea, nausea/vomiting, constipation and weight loss [1, 2]. As the disease progresses it leads to complications, such as fistulas, abscesses, strictures, perforation of the bowel and toxic megacolon [3, 4].

The exact etiology of IBD remains unknown, although environmental factors, the immune system, the microbiome and damage to the enteric nervous system (ENS) have been shown to influence its pathophysiology [2, 5, 6]. The ENS intrinsically innervates the GI tract and is responsible for monitoring and coordinating all aspects of gut function [7]. Clinical and experimental studies have demonstrated that intestinal inflammation is associated with loss of enteric neurons and destruction of nerve fibers [8–11], as well as functional changes in enteric neurons, including hyperexcitability and altered neurotransmission [12–15]. Furthermore, colonic inflammation affects changes in the neurochemical coding of myenteric neurons such as choline acetyltransferase (ChAT) and neuronal nitric oxide synthase (nNOS) neurons, causing disruption of GI functions [16–18]. Structural and functional changes in the ENS leading to alterations of intestinal functions can persist long after the resolution of acute intestinal inflammation [19, 20]. Damage to the ENS plays a role in the generation of IBD symptoms and underlies disease progression [21]. Infiltration of immune cells to the level of the myenteric and submucosal plexuses of the gut wall can be predictive of IBD recurrence [22, 23]. Together these findings suggest that targeting enteric neurons may be beneficial in combating IBD severity.

Currently, IBD treatments are focused towards suppressing inflammation with lifelong medications, such as antibiotics, corticosteroids, biologics and other immunomodulatory agents [24, 25]. Overall, current therapies have limited efficacy due to loss of patient’s responsiveness and severe side-effects [25]. Therefore, many IBD patients will undergo surgery for treatment of refractory disease or specific complications, often requiring repeated surgeries [25, 26]. Novel treatments for intestinal inflammation currently being investigated include the manipulation of gut microbiota and cellular therapies [27–29].

Mesenchymal stem cells (MSCs) have demonstrated significant potential for clinical use in a variety of inflammatory, autoimmune and nervous system diseases [30–32]. MSCs are multipotent cells that can be derived from many adult tissues and are capable of differentiating into multiple lineages within the appropriate microenvironment, including cells of neuronal and glial lineage [33–35]. Potential advantages of MSCs for cellular therapy include: ease of isolation, *in vitro* expansion capacity, low immunogenicity, receptiveness to *in vitro* genetic modification and a safe and feasible profile for transplantation into humans [36, 37]. Although not completely understood, it is considered that MSCs may exert therapeutic effects via secretion of biologically active molecules and factors that may have anti-inflammatory, immunomodulatory and neuroprotective effects [38–40]. Culture medium containing the MSC secretome released during *in vitro* expansion (MSC-conditioned medium (CM)) has demonstrated therapeutic potential in various pathologies [36].

Results from clinical trials in IBD patients with fistulae and luminal inflammation demonstrate that MSC therapy is both safe and feasible [27, 28, 41–43]. Experimental models of colitis have confirmed that MSCs migrate to sites of inflammation and exert their anti-inflammatory actions for the restoration of epithelial barrier integrity and repair of damaged tissue [44–48].

While the competence of MSCs to repair tissue damage associated with intestinal inflammation has been verified, there is currently only one study that has examined the effects of MSC-based therapies in attenuating inflammation-induced enteric neuropathy [18]. In this study, bone marrow-derived MSCs (BM-MSCs) and CM, administered by enema, were effective in protecting enteric neurons from 2,4,6-trinitrobenzene-sulfonate acid (TNBS)-induced inflammatory damage to the guinea-pig distal colon, as well as attenuating colonic dysmotility 7 days post treatment. Previous studies in animal models of multiple sclerosis, spinal cord injury, myocardial infarction, glaucoma and kidney injury reported that therapeutic effects of MSCs occur as early as several hours to several days after administration [49–53]. Furthermore, MSCs have been shown to exert neuroprotective, anti-inflammatory and immunomodulatory effects in experimental traumatic brain injury 2–3 days post administration [54, 55]. These studies suggest that long-term engraftment of MSCs is not required for their efficacy and that less than 7 days is sufficient to generate a therapeutic effect. While it was shown that MSCs alleviate enteric neuropathy associated with colitis 7 days after administration [18], the time point at which MSCs initiate their therapeutic effects has not been determined. Therefore, the aim of this study was to investigate at which time point following induction of colitis human BM-MSCs and CM become beneficial in protecting and repairing enteric neurons. This information will assist future investigations into the mechanisms of enteric neuroprotection by MSCs.

## Materials and Methods

### Animals

Male and female Hartley guinea-pigs weighing 140–280g (*n* = 69) were attained from South Australian Health and Medical Research Institute and randomly assigned to experimental groups. All guinea-pigs were housed in a temperature-controlled environment with 12 hour day/night cycles and free access to food and water. All animal experiments in this study complied with the guidelines of the National Health and Medical Research Council (NHMRC) Australian Code of Practice for the Care and Use of Animals for Scientific Purposes and were approved by the Victoria University Animal Experimentation Ethics Committee (ethics number AEETH12-012). All efforts were made to minimize animal suffering.

### MSC culture

Passage 4 human BM-MSC cell lines BM-7025 and BM-7081 (Tulane University) were characterized for their expression of surface antigens, differentiation potential, and colony forming ability as previously described [18]. All tests confirmed that MSCs used in this study met criteria for defining *in vitro* MSC cultures proposed by the International Society for Cellular Therapy (ISCT) [56]. Cells were plated at an initial density of 60 cells/cm^2^ and incubated in complete culture medium; minimum essential medium (α-MEM) supplemented with 16.5% MSC qualified fetal bovine serum (FBS) (validated by Life Technologies and is tested to successfully support the differentiation and culture of human MSCs according to ISCT guidelines), 100 U/mL penicillin/streptomycin, and 100X GlutaMAX (all purchased from GIBCO, Life Technologies) at 37°C. Medium was replenished every 48–72 hours for 10–14 days until the cells were 70–85% confluent (maximum). MSCs were rinsed in 5mL sterile phosphate buffered solution (PBS) (1X) prior to incubation with 3mL trypsin/ethylenediaminetetraacetic acid (EDTA) solution (TrypLE Select; GIBCO, Life Technologies) for 3 minutes at 37°C to detach cells. Enzymatic activity was neutralized by 8mL of stop solution (α-MEM + 5% FBS) and MSCs were collected and centrifuged at 450 g for 5 minutes at room temperature. Cells were then re-suspended in fresh culture medium and counted using a light microscope. CM was aspirated following a minimum of 48 hours incubation with MSCs and used for *in vivo* experiments. Unconditioned medium (UCM) is the culture medium described above, but not exposed to MSCs.

### Induction of TNBS colitis

For induction of colitis, 2,4,6-trinitrobenzene-sulfonic acid (TNBS) (Sigma) was dissolved in 30% ethanol to a concentration of 30mg/kg [15]. A total volume of 300μL was instilled by enema into the lumen of the colon through a lubricated silicone catheter approximately 7cm proximal to the anus. For TNBS administration, guinea-pigs were anesthetized with isoflurane (induced at 4%, maintained on 1–4% isoflurane in O_2_). Sham-treated guinea-pigs underwent the same procedure excluding TNBS administration.

### Treatment with MSCs, CM and UCM

Guinea-pigs in MSC-treated, CM-treated, and UCM-treated groups were anesthetized with isoflurane 3 hours after TNBS administration and administered 1x10^6^ MSCs, or 300μL CM, or 300μL UCM by enema into the colon via a silicone catheter. In hapten-induced colitis, ethanol serves to disrupt the mucosal barrier allowing TNBS to infiltrate the underlying colonic layers and initiate inflammation. The peak of ethanol-induced epithelial damage occurs at 3 hours [57], therefore this time point was selected for the administration of MSC-based therapies. Animals were held at an inverted angle following MSC-based treatments to prevent leakage from the rectum. Guinea-pigs were weighed and monitored daily following treatment. Animals were culled via stunning (a blow to the occipital region) and exsanguination [13, 14] 6, 24 and 72 hours after induction of colitis or sham treatment. Segments of the distal colon were collected for histological and immunohistochemical studies.

### Tissue preparation

Colon tissues were cut open along the mesenteric border, stretched and pinned flat with the mucosal side up for wholemount preparations; samples for cross sections were not stretched. Tissue samples were fixed overnight at 4°C in Zamboni’s fixative (2% formaldehyde and 0.2% picric acid) and subsequently washed in dimethyl sulfoxide (DMSO) (Sigma-Aldrich) and PBS to remove fixative. Samples for histology were fixed in 10% buffered formalin solution and stored in 70% ethanol until embedding.

### Immunohistochemistry

Immunohistochemistry (IHC) was performed on wholemount longitudinal muscle-myenteric plexus (LMMP) preparations and cross sections of the distal colon. For labelling of nerve fibers and immune cells, tissues were stored in 50:50 optimum cutting temperature (OCT) compound (Tissue-Tek) and sucrose solution for 24 hours at 4°C and subsequently frozen in liquid nitrogen-cooled isopentane and OCT compound. Samples were stored at -80°C until they were cryo-sectioned (30μm) onto glass slides for IHC. After incubation with 10% normal donkey serum (NDS) (Merck Millipore) for 1 hour at room temperature, sections were then labelled with primary antibodies: rabbit anti-β-tubulin (III) (1:1000) and mouse anti-CD45 (1:200) (both from Abcam) followed by secondary antibodies: donkey anti-rabbit Alexa Fluor 594 (1:200) and donkey anti-mouse FITC 488 (1:200) (both from Jackson Immunoresearch Laboratories). FITC-conjugated anti-human human leukocyte antigen (HLA)-A,B,C antibody (1:50) (BioLegend) was used to detect the presence of MSCs within the colonic wall. For labelling of myenteric neurons in wholemount LMMP preparations, tissues were dissected to expose the myenteric plexus by removing the mucosa, submucosa and circular muscle layers. After 1 hour incubation in 10% NDS at room temperature, wholemount preparations were labelled with primary antibodies: a pan neuronal marker mouse anti-HuC/HuD (1:500) (Merck Millipore), goat anti-neuronal nitric oxide synthase (nNOS) (1:500) (Novus Biologicals), goat anti-choline acetyltransferase (ChAT) (1:500) (Merck Millipore), mouse anti-CD45 (1:200), and rabbit anti-protein gene product 9.5 (PGP9.5) (1:500) (Abcam) followed by secondary antibodies: donkey anti-mouse Alexa Fluor 594 (1:200), donkey anti-goat FITC 488 (1:200), donkey anti-mouse FITC 488 (1:200), and donkey anti-rabbit Alexa Fluor 594 (1:200) (all from Jackson Immunoresearch Laboratories). All tissues were mounted on glass slides with fluorescent mounting medium (DAKO).

### Histology

After fixation, tissues were paraffin embedded, sectioned at 5μm, deparaffinized, cleared, and rehydrated in graded ethanol concentrations. For haematoxylin and eosin (H&E) staining, sections were immersed in Xylene (3x4 minutes), 100% ethanol (3 minutes), 90% ethanol, (2 minutes), 70% ethanol (2 minutes), rinsed in tap water, haematoxylin (4 minutes), rinsed in tap water, Scott’s tap water (1 minute), eosin (3 minutes), rinsed in tap water, 100% ethanol (2x1 minute), xylene (2x3 minutes) and mounted on glass slides with distrene plasticizer xylene (DPX) mountant. Gross morphological damage was blindly assessed by histological grading of three parameters: mucosal flattening (0 = normal, 3 = severe flattening), occurrence of hemorrhagic sites (0 = none, 3 = frequent sites), and variation of the circular muscle (0 = normal, 3 = considerable thickening of muscular layer) [13].

### Imaging

Confocal microscopy was performed on an Eclipse Ti confocal laser scanning system (Nikon). Fluorophores were visualized using a 488 nm excitation filter for Alexa 488 or FITC and a 559 nm excitation filter for Alexa 594 or Rhodamine Red. Z-series images were acquired at a nominal thickness of 0.5μm (512x512 pixels). The total number of myenteric neurons immunoreactive (IR) for Hu, nNOS, and ChAT, as well as CD45-IR cells were counted within eight randomly captured images (total area size 2mm^2^) per preparation at X60 magnification. In cross sections, the density of nerve fibers was determined by measuring β-tubulin (III)-IR per 2mm^2^ area with Image J software at X60 magnification. Gross morphological damage in H&E stained colon sections was visualized using an Olympus BX53 microscope (Olympus) and images were captured with CellSense^TM^ software.

### Gene expression analysis

Total RNA was extracted from 1x10^6^ BM-MSCs using a High Pure RNA Isolation Kit (Roche) as per the manufacturer's instructions. cDNA was prepared from 0.5μg RNA using a SuperScript III First-Strand Synthesis System Reverse Transcriptase-Polymerase Chain Reaction (RT-PCR) kit and oligo (dT) primers (Invitrogen) (S1 Table). PCR was performed with 1μL cDNA and primer pairs under the following cycling conditions: PCR products were resolved by electrophoresis in 2% agarose gels and the bands visualized under UV light.

### Flow cytometric cytokine analysis

MSCs were seeded at a density of 2×10^4^ cells/cm^2^ in 25cm^2^ filter cap flasks (Greiner Bio-One). After 48 hours in culture, samples of the media were collected to analyze the secretion of cytokines. Samples and cytokine standards with known concentrations were prepared according to the manufacturer’s instructions using the BD™ Cytometric Bead Array (BD Biosciences). Samples were incubated in the dark with cytokine capture beads and phycoerythrin (PE) detection reagent for 2 hours each before being transferred to fluorescence activated cell sorting (FACS, BD Biosciences) tubes; PE fluorescence was measured via a BD FACSCanto II flow cytometer with FACSDiva v6.1 software.

### Antibody array analysis

The RayBio® Label-based (L-Series) Human Antibody Array 1000 kit was used to determine presence of proteins secreted from MSCs and was performed following the manufacturer’s instructions (RayBiotech). Briefly, samples of equal protein concentrations were incubated with antibody membranes followed by incubation with biotin-conjugated anti-cytokine primary antibodies, as well as Cy3-conjugated streptavidin. Chemiluminescence was used for signal detection on a VersaDoc imaging system (Bio-Rad).

### Statistical analysis

Data were presented as mean ± standard error of the mean (SEM), if not specified otherwise. Statistical differences were determined by one-way ANOVA with Bonferroni *post hoc* test for multiple group comparisons using Prism v5.0 (GraphPad Software). Data were considered statistically significant when *P*<0.05.

## Results

### MSCs successively migrate transmurally and engraft at the site of inflammation

**Fig 1:**
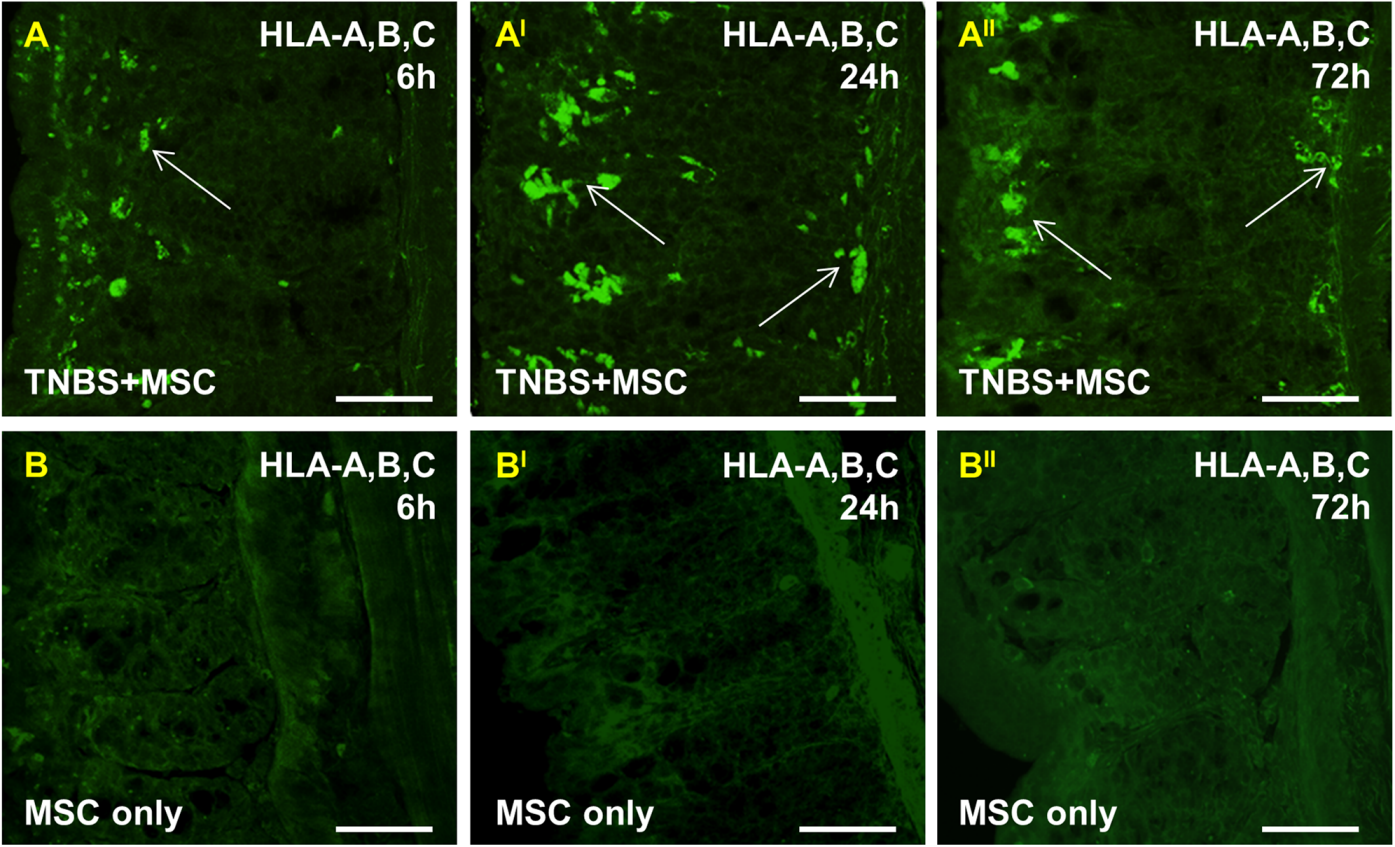
MSC homing within the inflamed colon. Migration and homing of human bone marrow MSCs to the inflamed area of the guinea-pig colon was confirmed using anti-HLA-A,B,C antibody specific to human MHC class I. MSCs administered 3 hours after TNBS application were localized mostly in the lamina propria 6 hours after induction of colitis **(A)**. Transmural engraftment of human MSCs into the colon wall to the level of the myenteric ganglia was evident 24 and 72 hours post induction of colitis **(A** ^**I**^ **-A** ^**II**^ **)**. At 6, 24 and 72 hours, HLA-A,B,C-IR cells were not detected in the distal colon from MSC-only treated guinea-pigs, validating absence of MSC homing to non-inflamed tissues **(B-B** ^**II**^ **)**. *n* = 3/group/time point. Scale bars = 50μm.

To assess the capacity of MSCs homing to the area of tissue damage and inflammation, segments of the distal colon were collected from guinea-pigs treated with 1) MSCs after TNBS and 2) MSCs only without TNBS. To identify adoptively transferred human MSCs within the guinea-pig colon, cross sections were labelled with anti-human HLA-A,B,C antibody to detect major histocompatibility complex class I (MHC class I) antigens which are expressed by all human nucleated cells (Fig 1). The successful engraftment of MSCs into the colonic wall was evident by localization of HLA-A,B,C positive cells in the colon sections collected at 6, 24 and 72 hours post induction of colitis (Fig 1A and 1A^II^). At 6 hours after TNBS administration, MSCs were present mostly in the mucosal lamina propria in colon sections from MSC-treated guinea-pigs (Fig 1A). At 24 and 72 hours after induction of colitis, transmural migration and engraftment of human MSCs into the colon wall to the level of the myenteric ganglia was evident (Fig 1A^I^ and 1A^II^). HLA A,B,C-IR cells were absent in colon sections from MSC-only administered animals at all time points indicating that MSC engraftment was not evident in non-inflamed tissues (Fig 1B and 1B^II^).

### MSC and CM treatments prevented weight loss in guinea-pigs with intestinal inflammation

**Fig 2:**
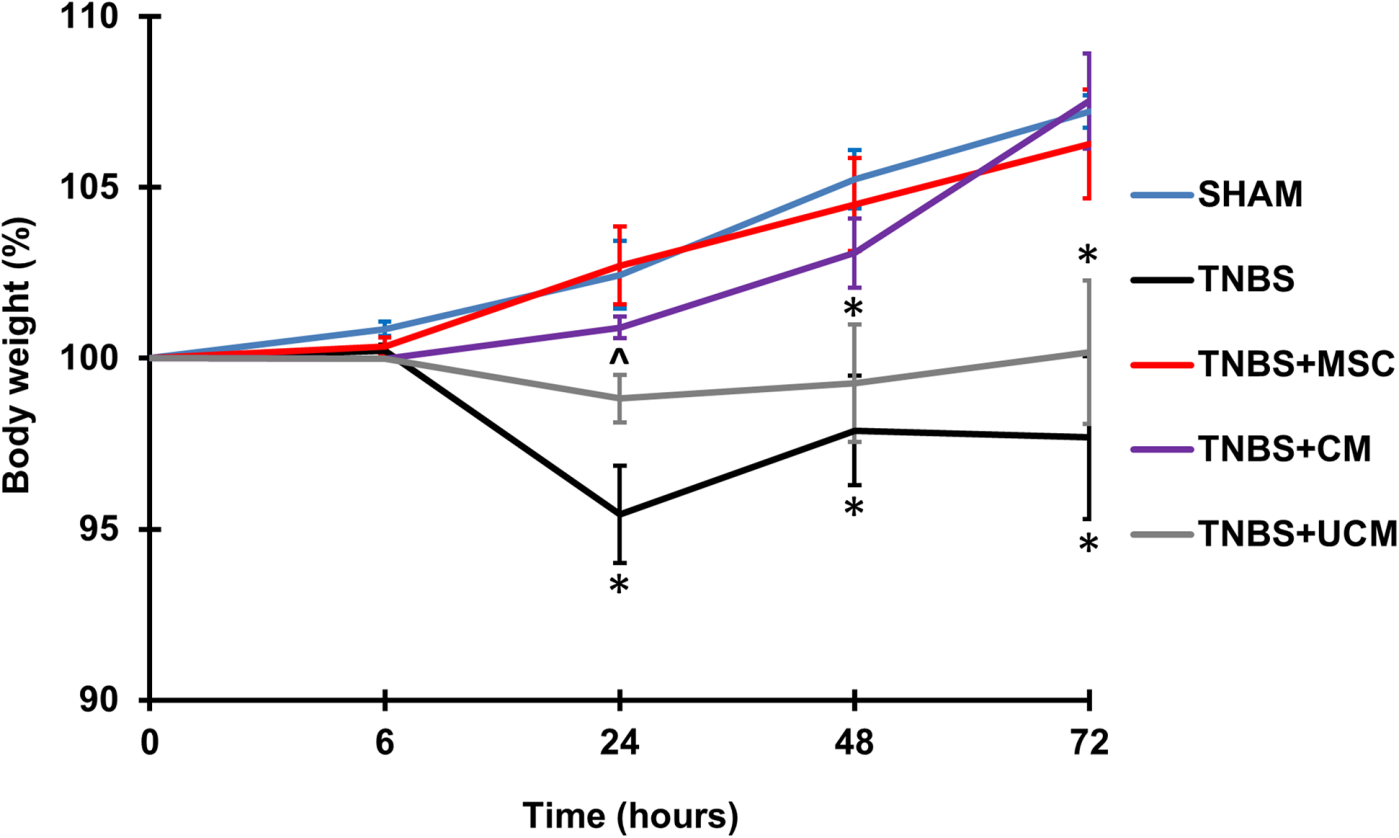
Body weight of guinea-pigs within 72 hours post induction of colitis or sham treatment. No change in guinea-pig weight was observed in all groups at the 6 hour time point. Within 72 hours, administration of TNBS-only and treatment with UCM induced significant loss of body weight, while treatment with MSCs and CM after the induction of colitis prevented loss of body weight. *n* = 4/group/time point. *P<0.05 compared to sham, MSC and CM-treated groups, ^*P*<0.05 compared to sham and MSC-treated groups.

To demonstrate the systemic impact of the tested MSC-based therapies, guinea-pig weight was monitored daily prior to and post induction of colitis and/or treatments (Fig 2, S2 Table). Sham-treated guinea-pigs consistently gained weight over 72 hours. At 6 hours after TNBS administration there was no change in weight between all groups. At 24, 48 and 72 hours after induction of inflammation, significant weight loss was observed in the TNBS groups and UCM groups compared to sham-treated animals (*P<*0.05). MSC and CM-treated animals consistently gained weight from 6 to 72 hours post induction of colitis similar to sham-treated guinea-pigs.

### MSC and CM treatments accelerated repair of damaged colonic architecture

**Fig 3:**
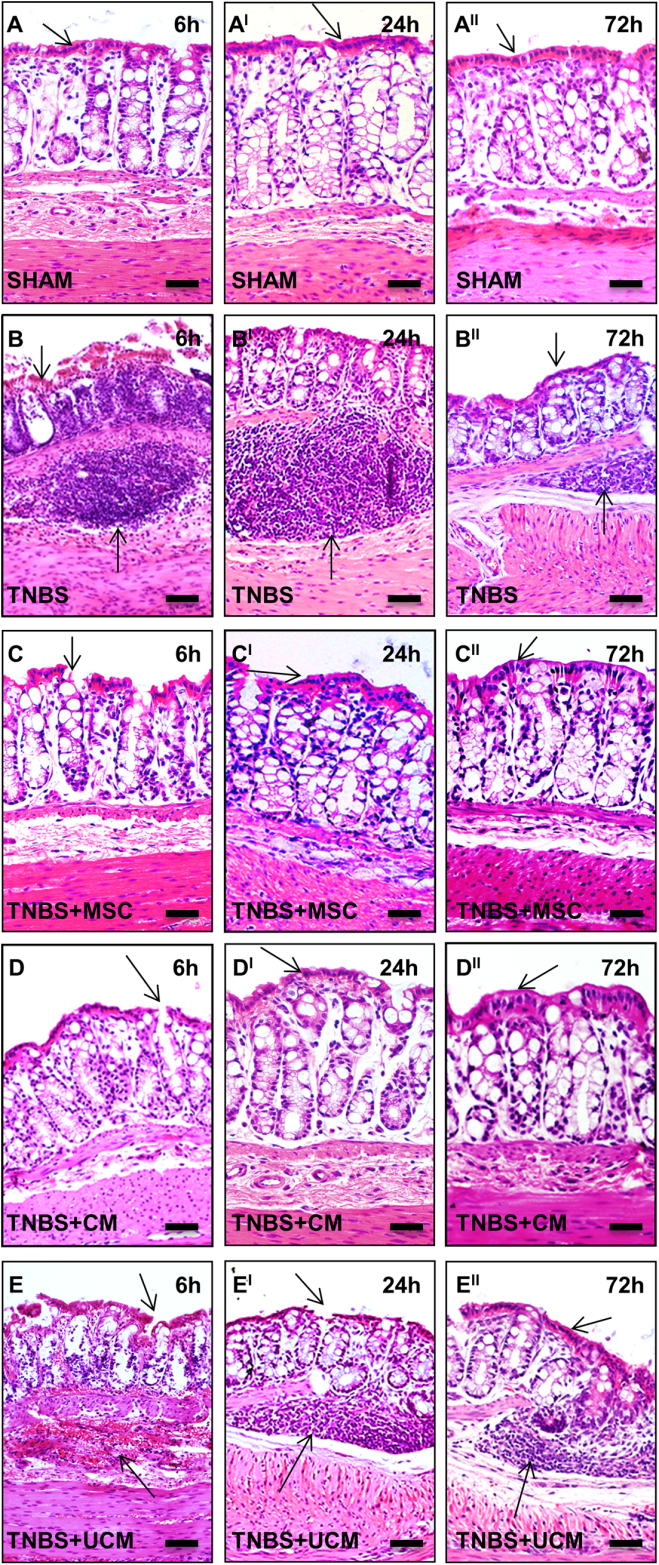
Gross morphological changes in the distal colon assessed in H&E stained cross sections. An intact epithelial lining and systematic arrangement of colonic layers were evident in sections from sham-treated animals at all time points **(A-A** ^**II**^ **)**. Immune infiltrate, flattening of the glands, disruption to the epithelial lining and destruction of mucosal epithelium, goblet cell loss and muscular edema were observed in colon tissues from TNBS-administered **(B-B** ^**II**^ **)** and UCM-treated **(E-E** ^**II**^ **)** guinea-pigs at all time points. Sections from MSC and CM-treated animals revealed initiation of colonic repair at 6 hours post induction of colitis **(C-D)**. Restoration of colonic architecture was observed at 24 **(C** ^**I**^ **-D** ^**I**^ **)** and 72 hours **(C** ^**II**^ **-D** ^**II**^ **)**. *n* = 4/group/time point. Scale bars = 50μm.

Gross morphological assessment of H&E-stained colon sections enabled definition of changes to colonic architecture 6, 24 and 72 hours after induction of TNBS-colitis (Fig 3). H&E-stained colon cross sections from sham-treated guinea-pigs displayed normal structural arrangements of goblet cells and crypts, a continuous epithelial cell lining and distinct colonic layers at 6, 24 and 72 hours post treatment (histological score 0–1) (Fig 3A–3A^II^). Conversely, examination of distal colon sections from guinea-pigs in the TNBS-only group showed immune cell infiltration and accumulation in the crypts and submucosal layers, goblet cell loss, glandular disruption and flattening, muscular edema and complete destruction of the epithelial cell lining at 6 hours (Fig 3B). Immune cell accumulation, flattening of glands and crypt distortion persevered 24 hours post induction of colitis (Fig 3B^I^). Some regeneration of the epithelium had occurred by 72 hours in TNBS-only administered animals; crypt morphology remained distorted, but infiltrates of immune cells were less prominent compared to 24 hours (Fig 3B^II^). Grading of gross morphological parameters in colon sections from TNBS-administered animals indicated histological score = 3 at 6 hours and score = 2–3 at 24 and 72 hours following induction of inflammation. Some disruption to glandular structure and epithelial cell lining was observed in colon sections from MSC and CM-treated guinea-pigs at 6 hours after induction of colitis (histological score = 1–2) (Fig 3C and 3D). However, mucosal healing and less flattening of crypts was evidenced when compared to sections from TNBS-only administered guinea-pigs. At 24 hours post induction of inflammation, sections from MSC and CM-treated animals revealed accelerated mucosal healing and repair to levels comparable with sham-treated animals (Fig 3C^I^–3D^I^). Furthermore, at the 72 hour time point, no evidence of mucosal damage and disruption to the colonic architecture in MSC-treated and CM-treated colon sections was present (histological score = 0–1) (Fig 3C^II^–3D^II^). Sections from UCM-treated animals displayed morphological damage similar to the TNBS-only group at 6 and 24 hour time points including immune cell infiltration, goblet cell loss, glandular distortion and flattening, muscular edema and destruction of the epithelial cell lining (histological score = 3) (Fig 3E and 3E^I^). After 72 hours, flattening of the glands and muscular edema was still evident similar to TNBS-only administered animals, however some signs of mucosal repair were observed (histological score = 2–3) (Fig 3E^II^).

### MSC and CM treatments attenuated the immune response in the distal colon 24 hours after induction of inflammation

**Fig 4:**
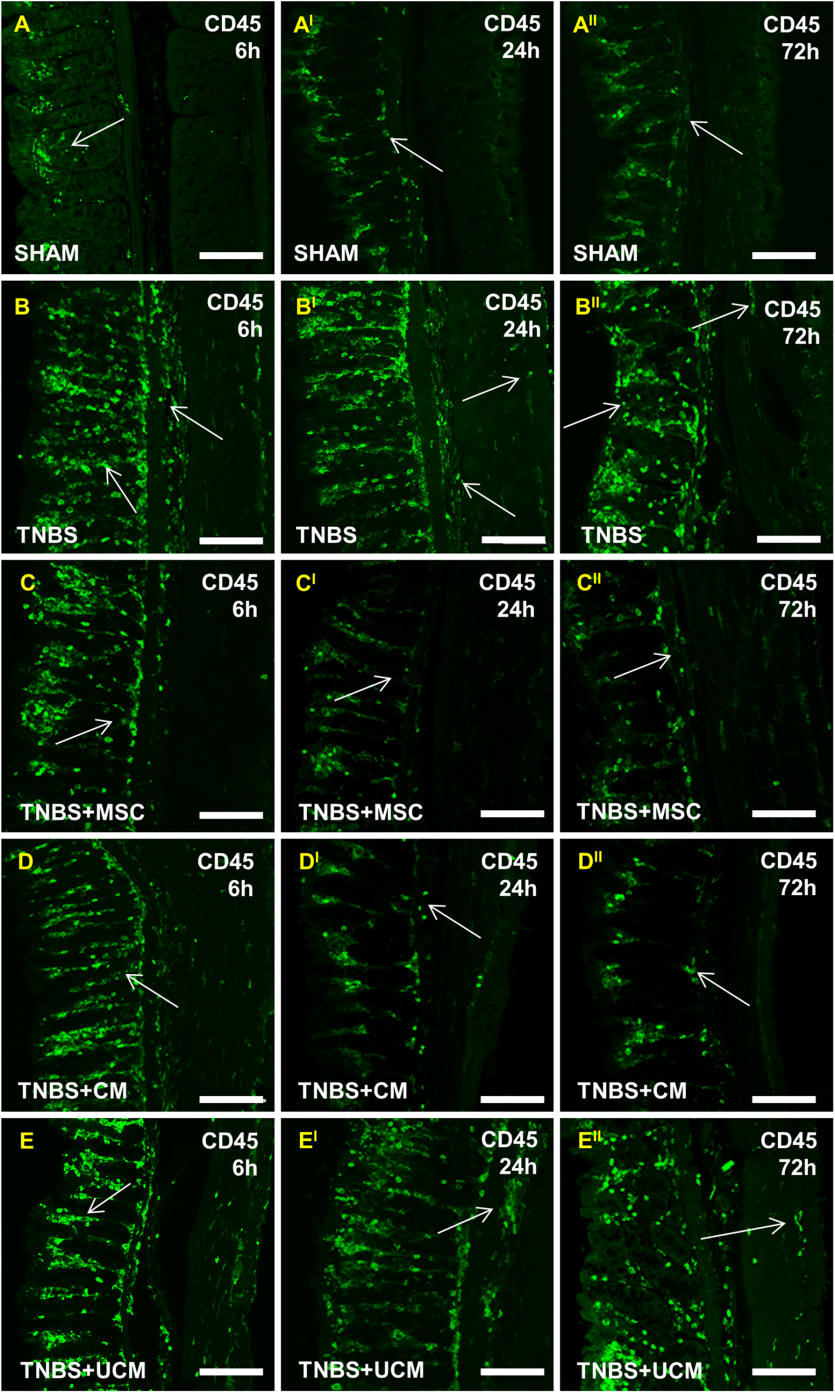
Leukocyte infiltration in colon cross sections. Cross sections of the distal colon were labelled with pan-leukocyte marker anti-CD45 antibody. At 6 hours, an increase in CD45-IR cells was observed in the mucosa of TNBS-only, MSC, CM and UCM-treated sections compared to sections from sham-treated animals **(A-E)**. At 24 and 72 hours, elevated numbers of CD45-IR cells were prominent transmurally through the layers of the colon wall in TNBS-only **(B** ^**I**^ **-B** ^**II**^ **)** and UCM-treated **(E** ^**I**^ **-E** ^**II**^ **)** cross sections. Treatment with MSCs and CM attenuated the increase of CD45-IR cells at 24 **(C** ^**I**^ **-D** ^**I**^ **)** and 72 hours **(C** ^**II**^ **-D** ^**II**^ **)**. *n* = 4/group/time point. Scale bars = 50μm.

**Fig 5:**
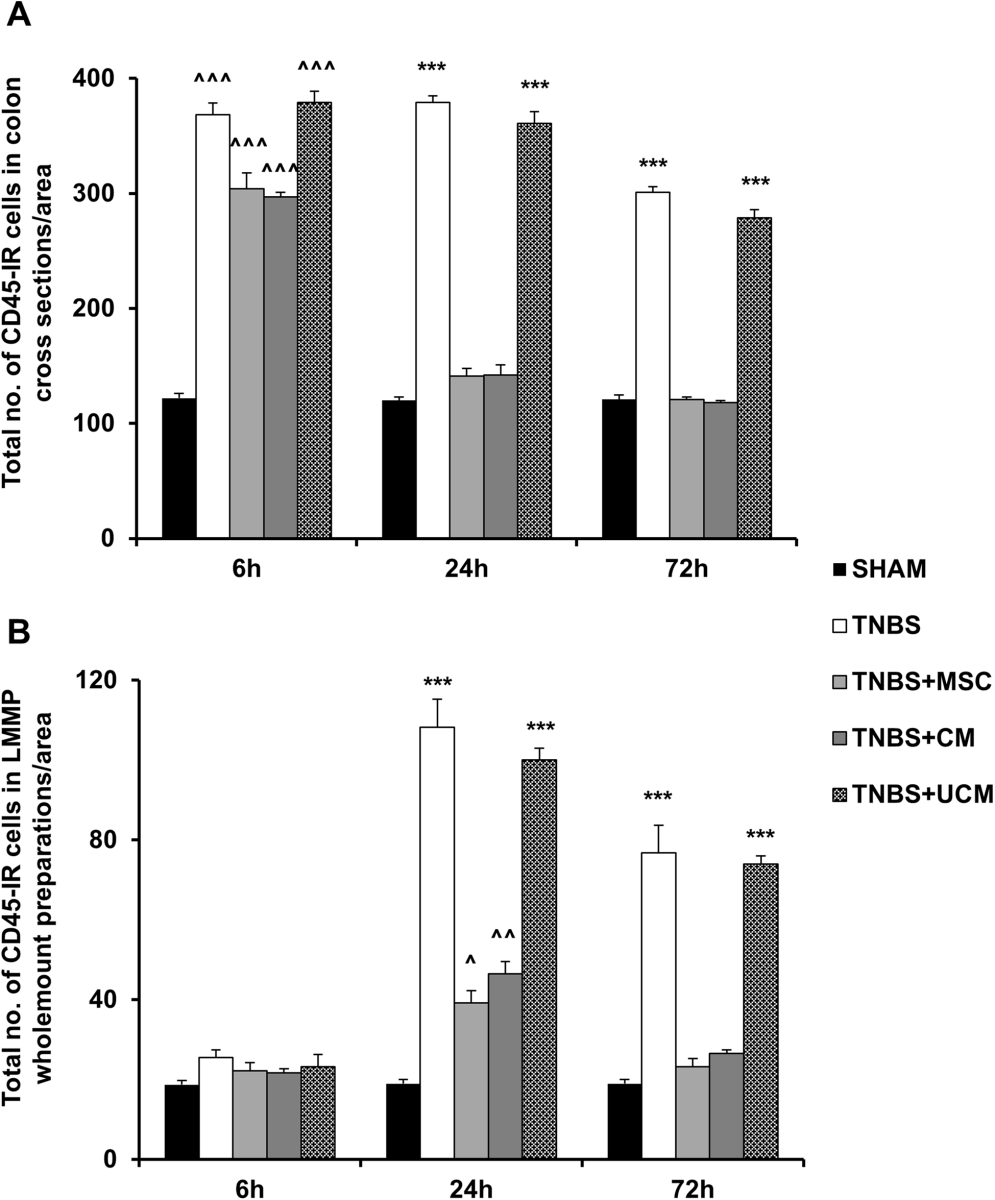
Quantitative analysis of leucocyte numbers in colon cross sections and wholemount preparations. Total number of leukocytes counted per 2mm^2^ area within the colon wall in cross sections **(A)**. Total number of leukocytes quantified per 2mm^2^ area in LMMP preparations **(B)**. *n* = 4/group/time point. ^*P* < .05, ^^*P* < .01, ^^^*P* < .001 compared to sham-treated group. ***P < .001 compared to sham, MSC and CM-treated groups.

To measure the severity of colitis and the anti-inflammatory efficacy of the treatments, quantitative analyses of CD45+ leukocytes were performed in colon cross sections and wholemount LMMP preparations. Considerably greater numbers of CD45-IR cells were present in the colon sections from TNBS-only (369±10 cells/area), MSC (304±14 cells/area), CM (297±4 cells/area) and UCM-treated (379±10 cells/area) animals compared to sham-treated (122±4 cells/area) animals at the 6 hour time point (*P<*0.001 for all, Figs 4A–4E and 5A). At 24 hours, the numbers of CD45-IR cells were reduced in colon sections from MSC (141±7 cells/area) and CM-treated (142±9 cells/area) guinea-pigs and were similar to the number of CD45-IR cells in sections from sham-treated (120±3 cells/area) animals. The numbers of leukocytes in colon sections from TNBS-only (379±6 cells/area) and UCM-treated (361±10 cells/area) animals remained elevated compared to sham, MSC and CM-treated groups (*P<*0.001 for all; Figs 4A^I^–4E^I^ and 5A). CD45-IR cells were identified throughout the thickness of the colon in sections from TNBS-only and UCM-treated animals compared to sections from other groups. The numbers of CD45-IR cells in sections from TNBS-only (301±5 cells/area) and UCM-treated (279±7 cells/area) guinea-pigs were slightly reduced by 72 hours, but were still higher compared to the number of leukocytes in the sections from sham (121±4 cells/area), MSC (121±2 cells/area) and CM-treated (118±2 cells/area) guinea-pigs (*P<*0.001 for all, Figs 4A^II^–4E^II^ and 5A).

**Fig 6:**
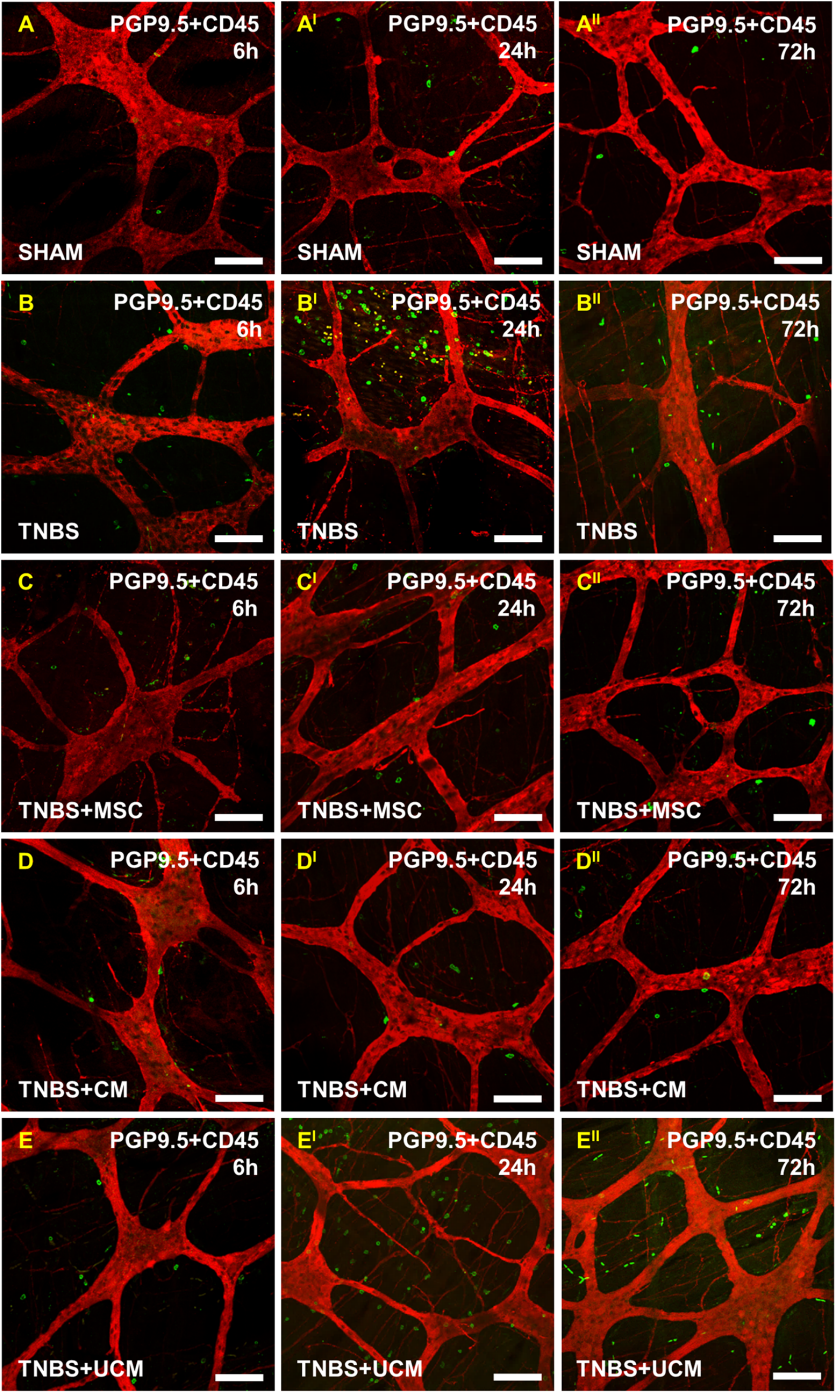
Infiltration of leukocytes to the level of the myenteric plexus post induction of colitis. CD45-IR cells in LMMP colon preparations. At 6 hours, a low level of leukocyte infiltration around the myenteric ganglia was observed in all groups **(A-E)**. At 24 hours, leukocyte infiltration in LMMP preparations from TNBS-only **(B** ^**I**^ **)** and UCM-treated **(E** ^**I**^ **)** animals was elevated in comparison to sham **(A** ^**I**^ **)**, MSC **(C** ^**I**^ **)** and CM-treated **(D** ^**I**^ **)** groups. CD45-IR cells were still evident at 72 hours in preparations from TNBS-only **(B** ^**II**^ **)** and UCM-treated **(E** ^**II**^ **)** animals. Leukocytes in preparations from MSC and CM-treated groups returned to sham-treated levels by 72 hours **(C** ^**II**^ **-D** ^**II**^ **)**. *n* = 4/group/time point. Scale bars = 100μm.

Immune cell infiltration to the level of myenteric ganglia was quantified in wholemount LMMP preparations by double labelling with anti-CD45 antibody and pan-neuronal marker anti-PGP9.5 antibody (Fig 6). At 6 hours, quantitative analysis of immune cells per 2mm^2^ area showed similar numbers of CD45-IR cells in LMMP preparations of the colon from sham (19±1 cells/area), TNBS-only (26±3 cells/area), MSC (22±2 cells/area), CM (22±1 cells/area), and UCM-treated animals (23±3 cells/area, Fig 5B). After 24 hours, the number of leukocytes at the level of the myenteric plexus was greater in tissues from TNBS-only (108±7 cells/area) and UCM-treated (100±3 cells/area) animals compared to sections from sham (19±1 cells/area), MSC (39±3 cells/area) and CM-treated (47±3 cells/area) guinea-pigs (*P<*0.001 for all; Figs 5B and 6A^I^–6E^I^). However, the numbers of CD45+ cells in colon preparations from MSC and CM-treated animals were higher than in preparations from sham-treated guinea-pigs (MSC: *P<*0.05, CM: *P<*0.01). By 72 hours after induction of inflammation, the numbers of immune cells at the level of the myenteric ganglia in colon preparations from MSC (23±2 cells/area) and CM-treated (27±1 cells/area) animals were comparable to the number of immune cells in preparations from sham-treated (19±1 cells/area) guinea-pigs. The quantity of CD45+ cells in preparations of the distal colon from TNBS-only (77±7 cells/area) and UCM-treated (74±2 cells/area) animals at 72 hours was higher when compared to sham, MSC, and CM-treated guinea-pigs (*P<*0.001 for all, Figs 5B and 6A^II^–6E^II^).

### MSC and CM treatments facilitated re-growth of nerve fibers and protected against neuronal loss 24 hours after induction of colitis

**Fig 7:**
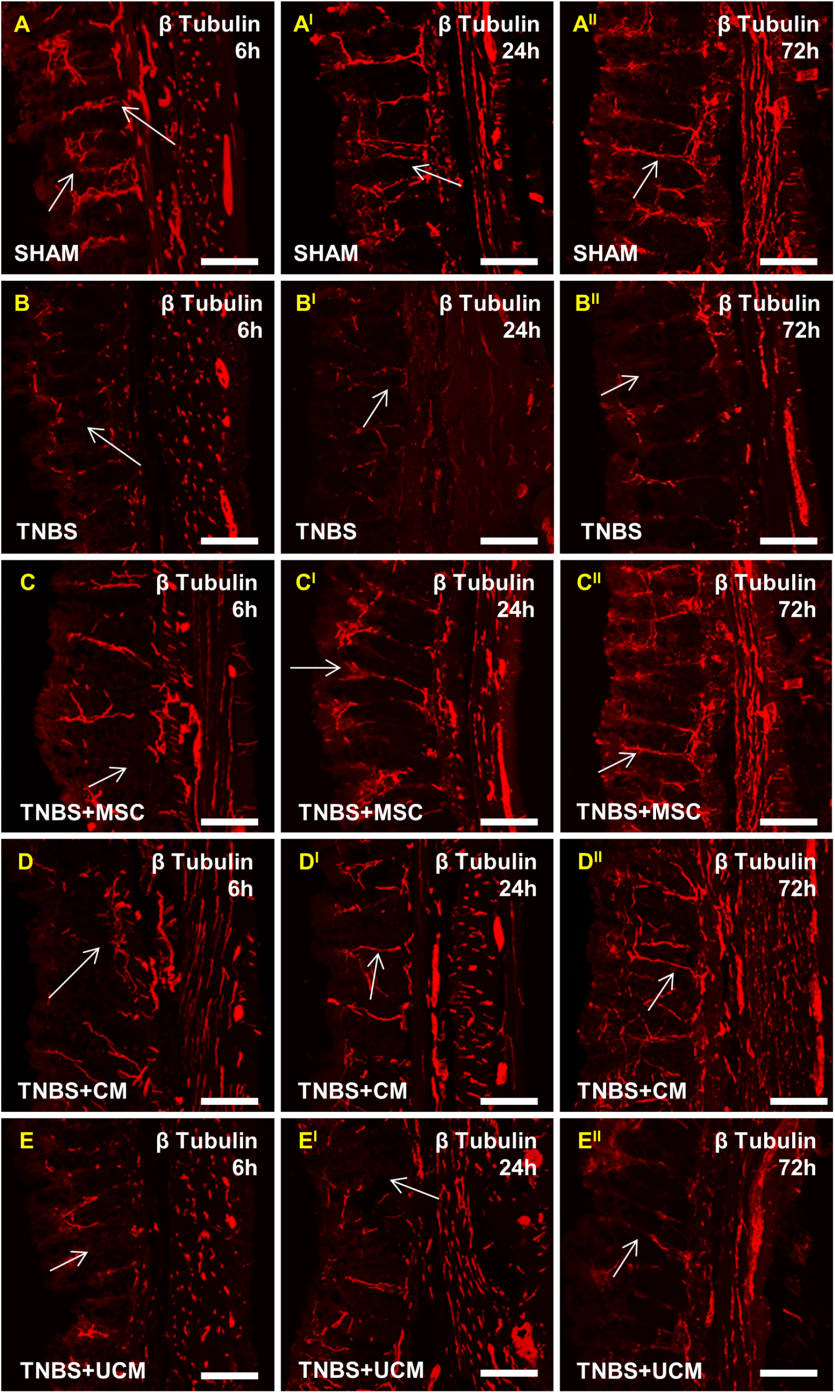
Nerve fibers in cross sections of the distal colon. Orderly distribution of fibers labelled by neuron specific anti-β-tubulin (III) antibody was observed in colon sections from sham-treated guinea-pigs at all time points **(A-A** ^**II**^ **)**. Disorganized, fragmented, and irregularly dispersed fibers were evident within the mucosa of sections from TNBS-only **(B-B** ^**II**^ **)** and UCM-treated **(E-E** ^**II**^ **)** animals at all time points. Damage to nerve fibers was present in sections from MSC and CM-treated animals at 6 hours **(C-D)**, however re-growth of nerve fibers was observed at 24 **(C** ^**I**^ **-D** ^**I**^ **)** and 72 hours **(C** ^**II**^ **-D** ^**II**^ **)**. *n* = 4/group/time point. Scale bars = 50μm.

**Fig 8:**
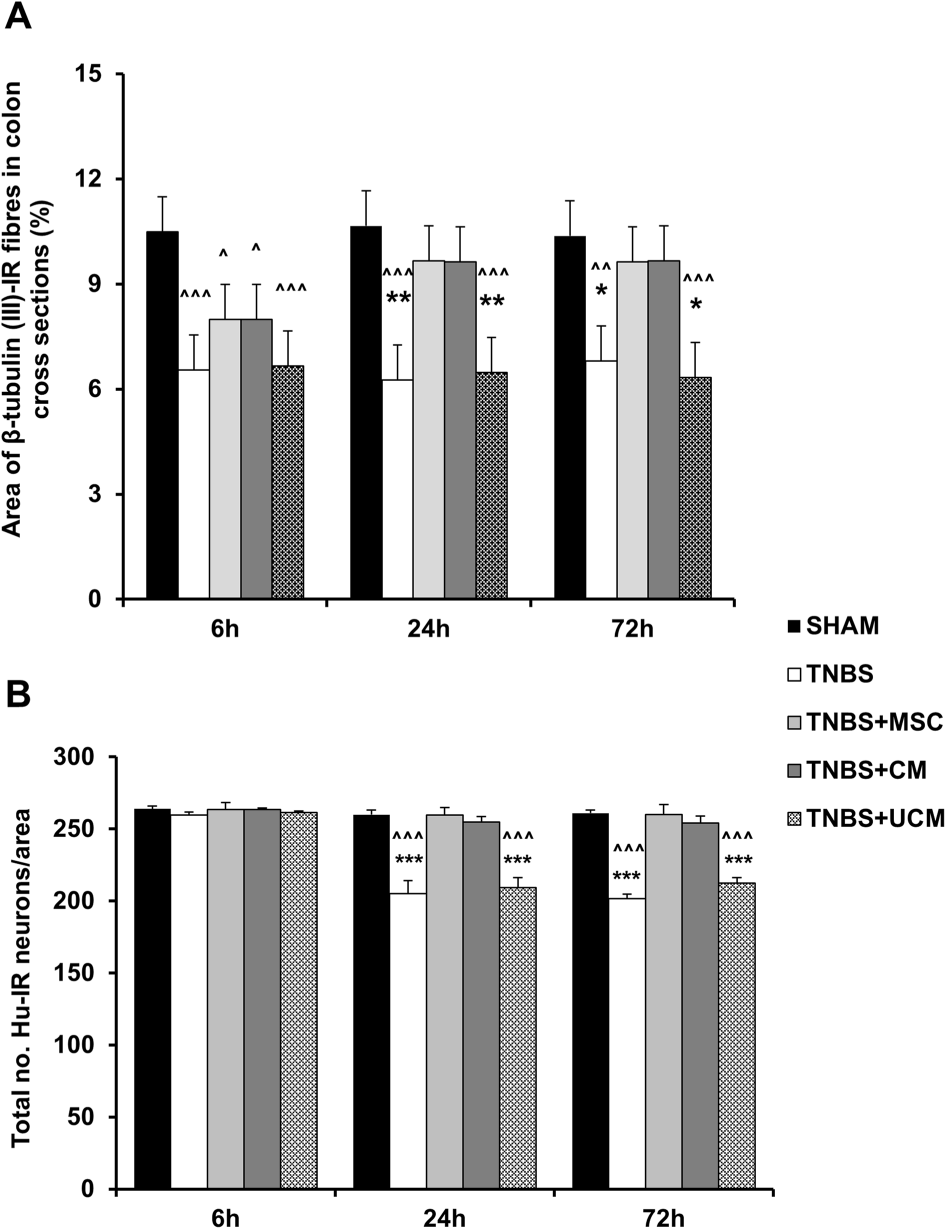
Quantitative analysis of nerve fiber density and the total number of neurons in the myenteric ganglia. Nerve fiber density quantified per 2mm^2^ area in cross sections of the distal colon **(A)**. The total number of myenteric neurons per 2mm^2^ area in LMMP wholemount preparations **(B)**. *n* = 4/group/time point. ^*P* < .05, ^^*P* < .01, ^^^*P* < .001 compared to sham-treated group. *P < .05, **P < .01, ***P < .001 compared to MSC and CM-treated groups.

Cross sections of the guinea-pig distal colon were labelled with antibody specific to neuronal microtubule protein β-tubulin (III) which identifies neuronal cell bodies and processes innervating smooth muscles and mucosa (Fig 7). Orderly distribution of β-tubulin (III)-IR fibers within the mucosal gland cores, submucosal and muscular layers were observed in colon sections from sham-treated guinea-pigs at all time points (Fig 7A–7A^II^). Quantification of fiber density demonstrated the total area of β-tubulin (III)-IR fibers in sections from sham-treated animals was 10.5±1.4%, 10.7±1.3% and 10.4±1.4% at 6, 24 and 72 hours after treatment, respectively (Fig 8A). At all time points following TNBS administration, β-tubulin (III)-IR fibers in colon sections from animals in the TNBS-only and UCM-treated groups were disorganized, fragmented, and dispersed irregularly within the mucosa (Fig 7B–7B^II^ and 7E–7E^II^). Compared to sections from sham-treated animals, quantitative analysis revealed lower β-tubulin (III)-IR fiber density at all time points in colon sections from TNBS-only (6 hours: 6.6±1.3%, *P<*0.001, 24 hours: 6.3±1.4%, *P<*0.001, 72 hours: 6.8±1.4%, *P<*0.01) and UCM-treated (6 hours: 6.6±1.4%, 24 hours: 6.5±1.3%, 72 hours: 6.3±1.5%, *P<*0.001 for all) guinea-pigs (Fig 8A). Treatment with MSCs and CM did not prevent damage to the nerve fibers 6 hours after TNBS administration; the density and organization of fibers in sections from MSC and CM-treated animals were similar to sections from TNBS-only and UCM-treated guinea-pigs (Fig 7C–7D^II^). Subsequently, the density of β-tubulin (III)-IR fibers in sections from MSC (7.9±1.3%) and CM-treated (7.9±1.4%) animals at the 6 hour time point was less when compared to sham-treated animals (*P<*0.05 for both, Fig 8A). This result suggests that 6 hours is insufficient time for the regenerative effect of MSC-based treatments to occur. The density of β-tubulin (III)-IR fibers in colon sections from MSC and CM-treated guinea-pigs were comparable to those in sections from sham-treated animals at the 24 (MSC: 9.7±1.4%, CM: 9.6±1.4%) and 72 hour time points (MSC: 9.6±1.4%, CM: 9.7±1.4%) (Figs 7C^I^–7D^II^ and 8A). β-Tubulin (III)-IR fiber density in colon sections from MSC and CM-treated guinea-pigs was higher when compared to sections from TNBS-only and UCM-treated animals at 24 (*P<*0.01 for both) and 72 hours (*P<*0.05 for both), but not after 6 hours (Fig 8A). Thus, MSC and CM treatments facilitated fiber regeneration and axonal re-growth 24 and 72 hours after induction of inflammation.

**Fig 9:**
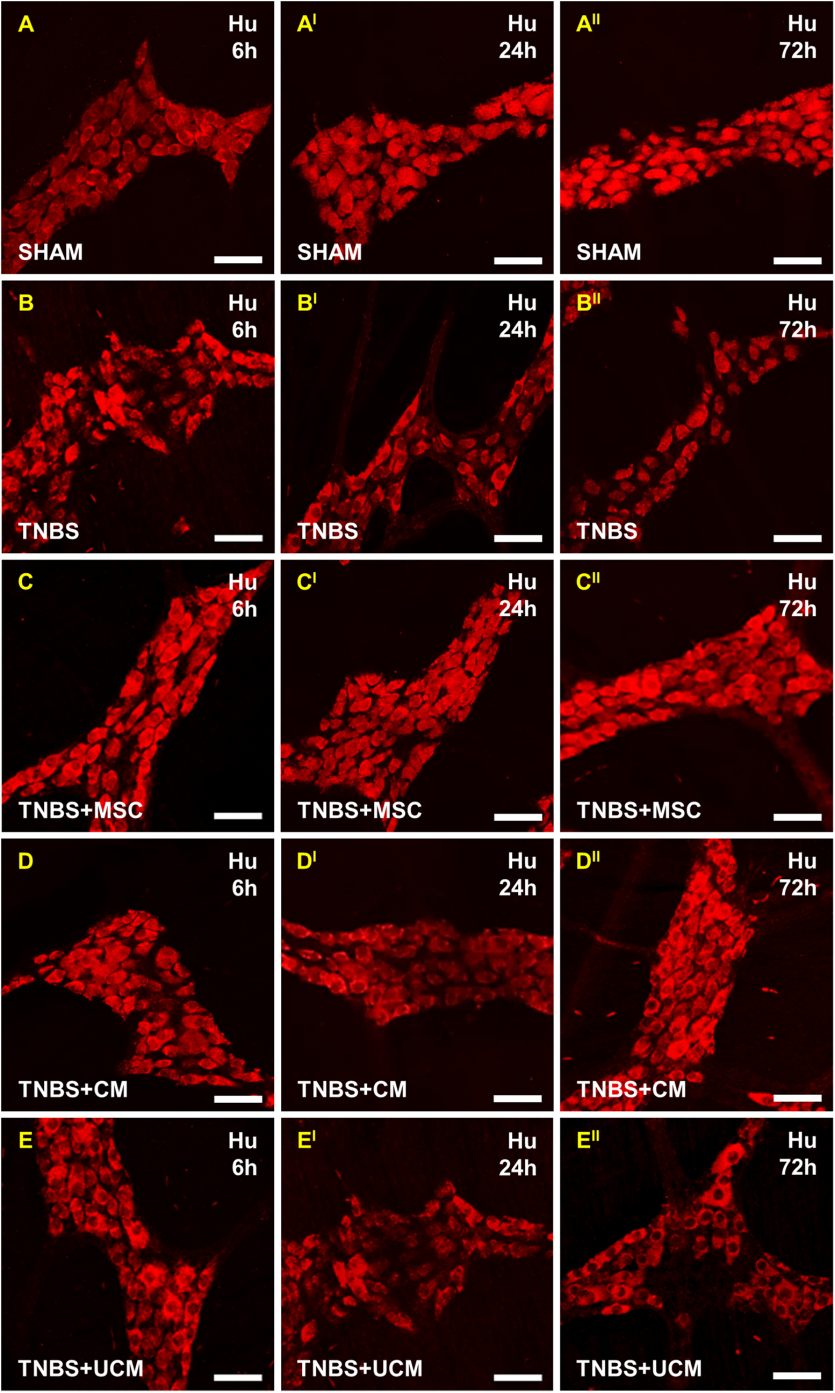
Effects of MSC and CM treatments on the total number of myenteric neurons. Myenteric neurons were identified by anti-Hu antibody in wholemount preparations of the distal colon. At 6 hours post TNBS administration, the number of myenteric neurons was similar across all groups **(A-E)**. At 24 and 72 hours, significant loss of neurons was observed in sections from TNBS-only **(B** ^**I**^ **-B** ^**II**^ **)** and UCM-treated **(E** ^**I**^ **-E** ^**II**^ **)** compared to sections from sham-treated **(A** ^**I**^ **-A** ^**II**^ **)** animals, but not in MSC **(C** ^**I**^ **-C** ^**II**^ **)** and CM-treated **(D** ^**I**^ **-D** ^**II**^ **)** groups. *n* = 4/group/time point. Scale bars = 50μm.

To study the effects of MSC and CM treatments on the number of myenteric neurons, neuronal cell bodies were labelled with the pan-neuronal marker anti-Hu antibody in wholemount LMMP preparations of the distal colon (Fig 9). The number of Hu-IR neurons counted within a 2mm^2^ area did not differ between all groups at 6 hours (sham: 264±2 cells/area, TNBS: 260±3 cells/area, MSC: 264±2 cells/area, CM: 264±1 cells/area, UCM: 262±1 cells/area) (Figs 8B and 9A–9E). At 24 hours after induction of colitis, the number of Hu-IR myenteric neurons decreased in colon preparations from TNBS-only (205±9 cells/area) and UCM-treated (209±7 cells/area) guinea-pigs compared to sham-treated animals (260±2 cells/area, *P<*0.001 for both). MSC and CM treatments attenuated neuronal loss associated with intestinal inflammation at 24 hours (MSC: 260±3 cells/area, CM: 255±4 cells/area) (Figs 8B and 9A^I^–9E^I^). At 72 hours, a reduction in the number of Hu-IR neurons was observed in LMMP preparations of the distal colon from TNBS-only (202±5 cells/area) and UCM-treated (212±5 cells/area) guinea-pigs compared to preparations from sham (261±4.9 cells/area), MSC (260±7 cells/area), and CM-treated (254±5 cells/area) animals (*P<*0.001 for all, Figs 8B and 9A^II^–9E^II^).

### Effects of MSC and CM treatments on inhibitory and excitatory neurons in the distal colon

**Fig 10:**
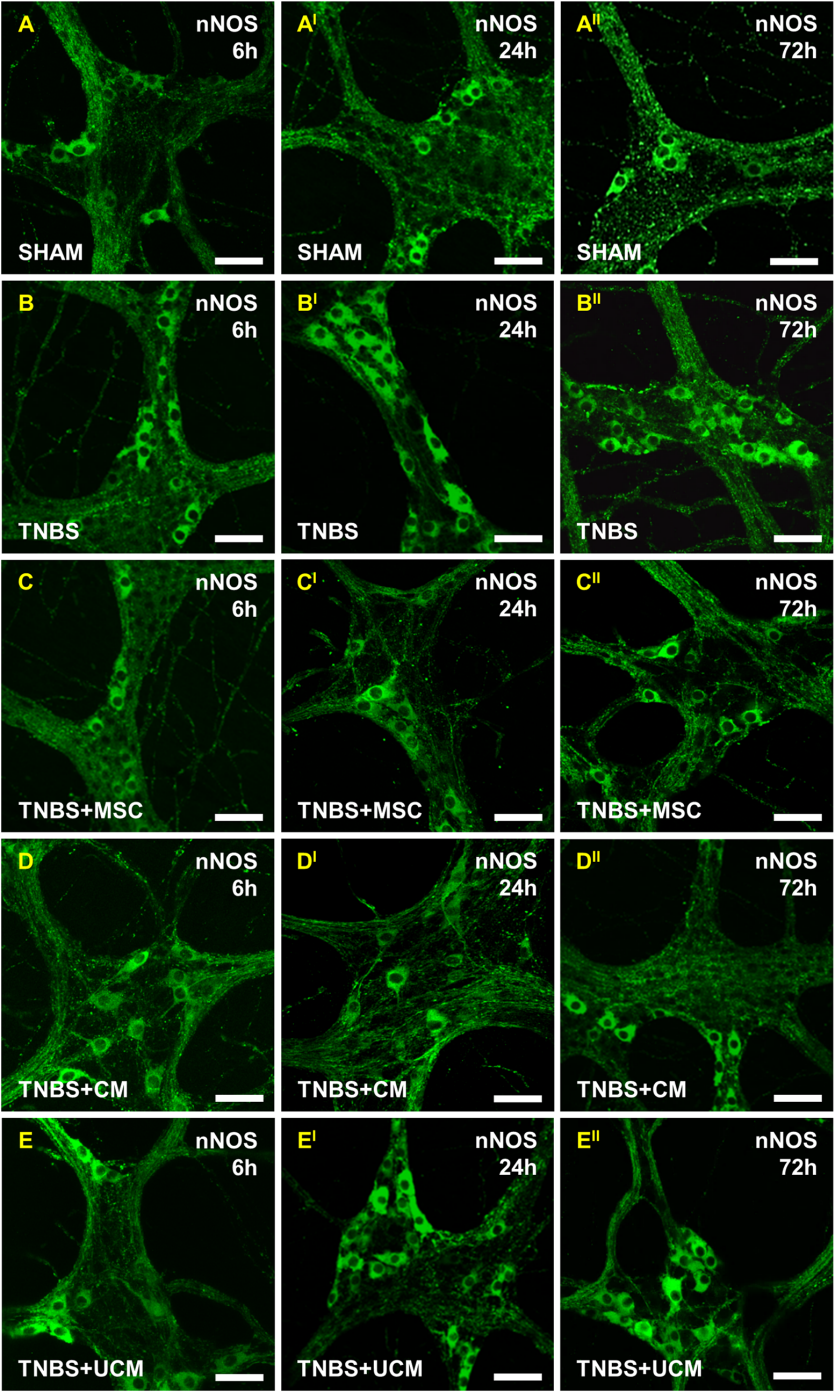
Effects of MSC and CM treatments on nNOS-IR myenteric neurons. No changes in nNOS-IR neurons were observed at 6 hours in any group **(A-E)**. At 24 and 72 hours, an increase in nNOS-IR neurons was observed in sections from TNBS-only **(B** ^**I**^ **-B** ^**II**^ **)** and UCM-treated **(E** ^**I**^ **-E** ^**II**^ **)** animals compared to sham **(A** ^**I**^ **-A** ^**II**^ **)**, but not in MSC and CM-treated groups **(C** ^**I**^ **-C** ^**II**^ **, D** ^**I**^ **-D** ^**II**^ **)**. *n* = 4/group/time point. Scale bars = 50μm.

**Fig 11:**
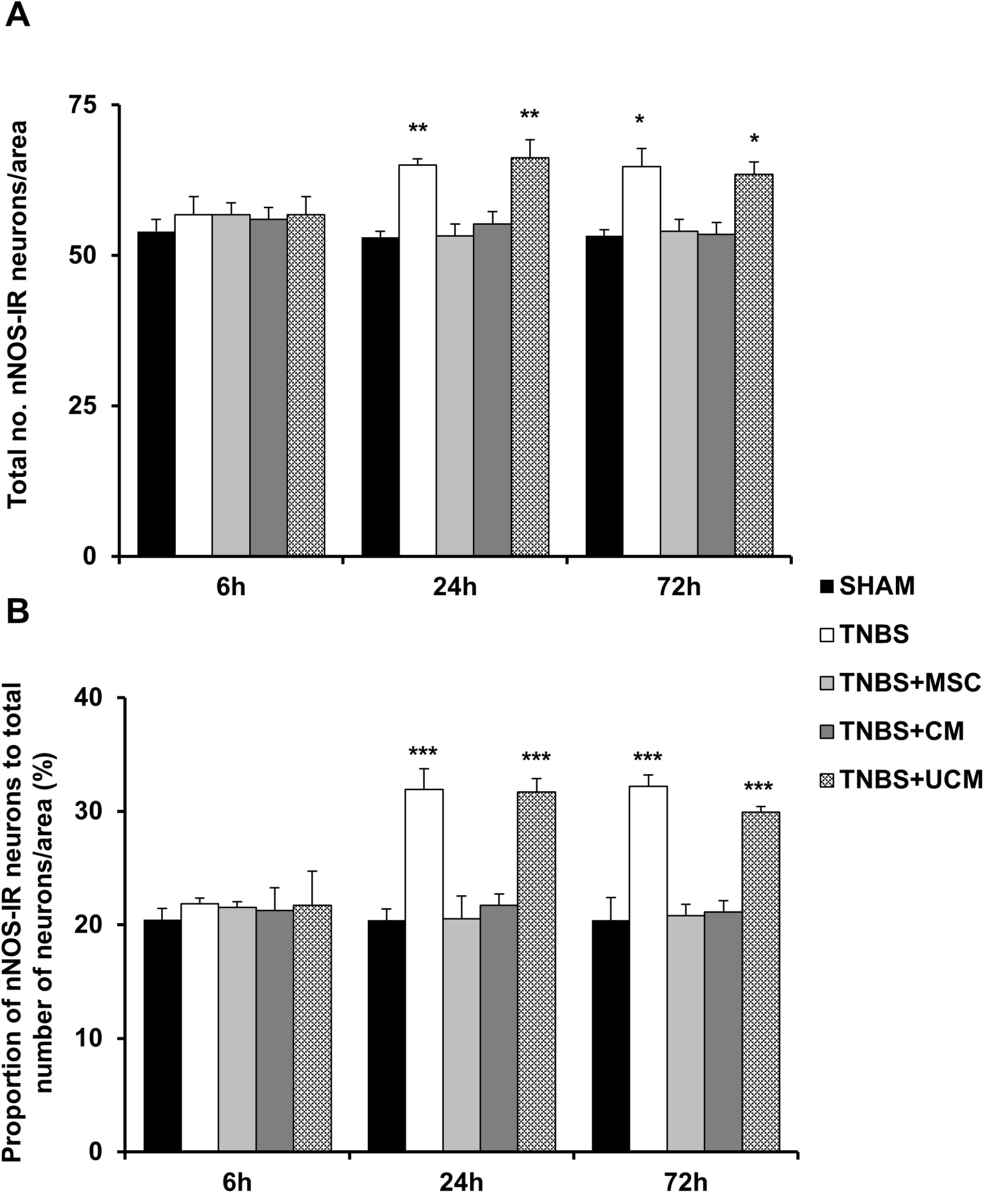
Quantification of the number and proportion of nNOS-IR myenteric neurons. The total number of nNOS-IR neurons identified by anti-nNOS antibody per 2mm^2^ area **(A)**. The proportion of nNOS-IR neurons to the total number of neurons **(B)**. *n* = 4/group/time point. *P < .05, **P < .01, ***P < .001 compared to sham, MSC and CM-treated groups.

Changes in inhibitory and excitatory myenteric neurons underlie inflammation-induced colonic dysmotility (16–18); therefore we investigated the effects of MSC-based therapies on these neurons. Inhibitory neurons were identified by using nNOS immunoreactivity in wholemount LMMP preparations of the distal colon (Fig 10). The number and proportion of nNOS-IR neurons were comparable between all groups at the 6 hour time point (sham: 54±2 cells/area, 20±1%, TNBS: 57±3 cells/area, 22±1%, MSC: 57±2 cells/area, 22±2%, CM: 56±2 cells/area, 21±2%, UCM: 57±3 cells/area, 22±3%, Figs 10A–10E and 11). At 24 hours, the quantity of nNOS-IR myenteric neurons increased in the colon in TNBS-only (65±1 cells/area) and UCM-treated (66±3 cells/area) guinea-pigs compared to sham (53±1 cells/area), MSC (53±2 cells/area) and CM-treated (55±2 cells/area) animals (*P<*0.001 for all; Figs 10A^I^–10E^I^ and 11A). The proportion of nNOS-IR neurons to the total number of neurons 24 hours post induction of colitis was greater in TNBS-only (31.9±1.8%) and UCM-treated (31.7±1.2%) guinea-pigs compared to sham (20±0.5%), MSC (21±1%), and CM-treated (22±1%) animals (*P<*0.001 for all; Fig 11B). At 72 hours, the number of nNOS-IR neurons in preparations of the distal colon from TNBS-only (65±3 cells/area) and UCM-treated (64±2 cells/area) guinea-pigs was higher compared to sham (53±1 cells/area), MSC (54±2 cells/area) and CM-treated (54±2 cells/area) animals (*P<*0.05 for all; Figs 10A^II^–10E^II^ and 11A). The proportion of nNOS-IR neurons at 72 hours was greater in colon preparations from TNBS-only (32±2%) and UCM-treated (30±0.5%) guinea-pigs compared to sham (20±0.5%), MSC (21±1%), and CM-treated (21±1%) animals (*P<*0.001 for all; Fig 11B).

**Fig 12:**
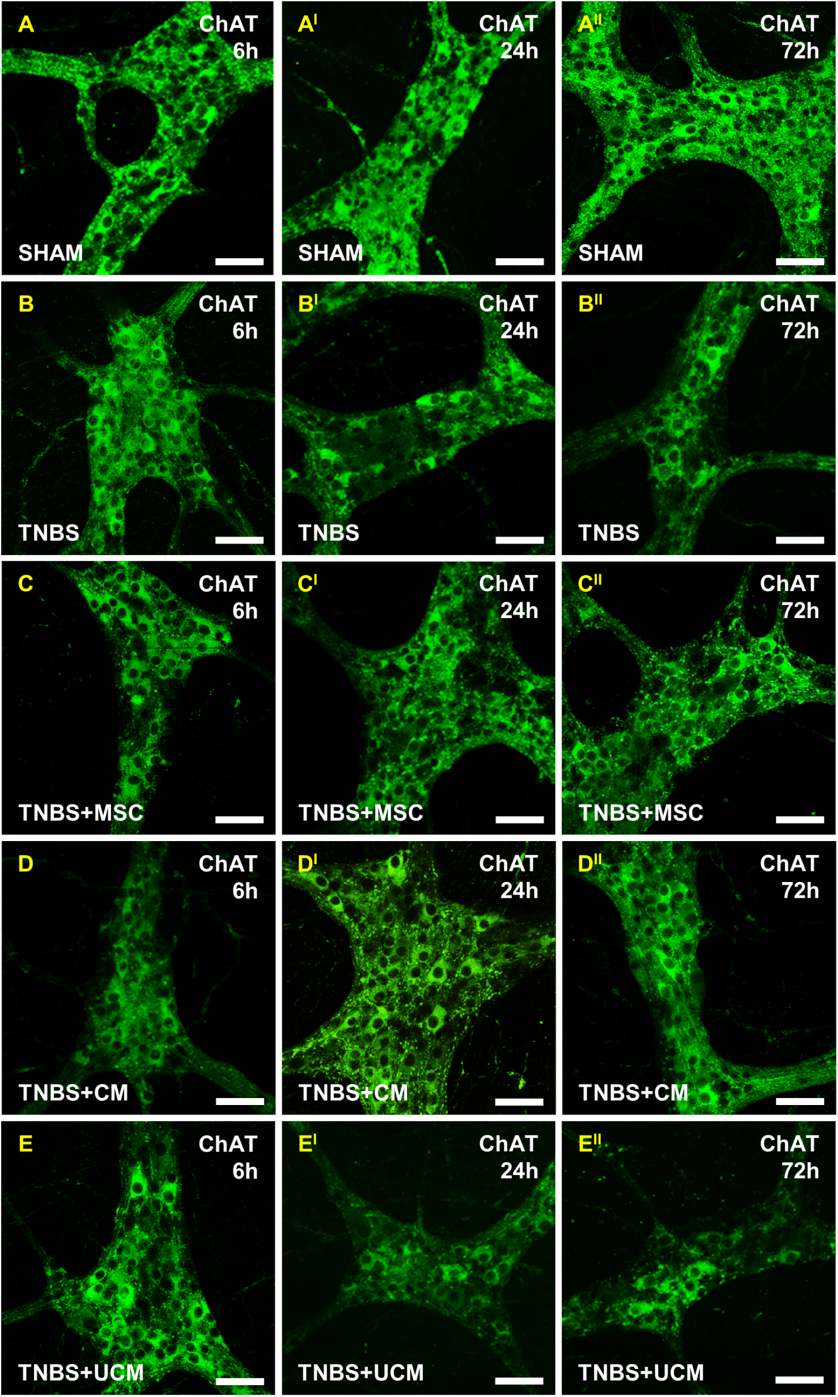
Effects of MSC and CM treatments on ChAT-IR myenteric neurons. No changes in the number of ChAT-IR myenteric neurons was observed in any group at 6 hours **(A-E)**. Significant loss of ChAT-IR neurons was observed in sections from TNBS-only and UCM-treated animals at 24 and 72 hours **(B** ^**I**^ **-B** ^**II**^ **, E** ^**I**^ **-E** ^**II**^ **)**. The number of ChAT-IR myenteric neurons was unaffected in MSC and CM-treated groups at 24 and 72 hours **(C** ^**I**^ **-C** ^**II**^ **, D** ^**I**^ **-D** ^**II**^ **)**. *n* = 4/group/time point. Scale bars = 50μm.

**Fig 13:**
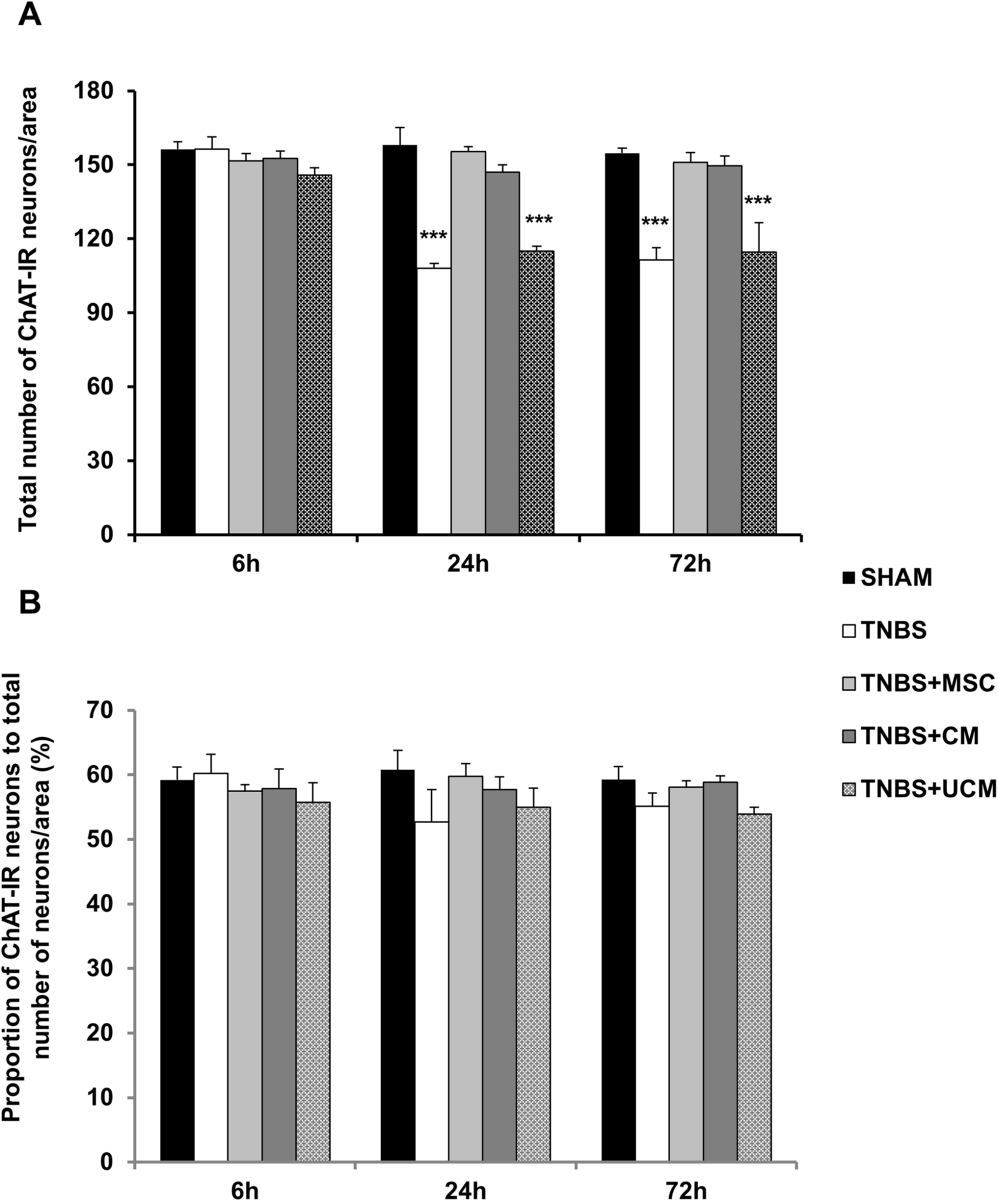
Quantification of the number and proportion of ChAT-IR myenteric neurons. The total number of ChAT-IR neurons quantified per 2mm^2^ area **(A)**. The proportion of ChAT-IR neurons to the total number of neurons **(B)**. *n* = 4/group/time point. ***P < .001 compared to sham, MSC and CM-treated groups.

Excitatory neurons were identified using ChAT immunoreactivity in wholemount preparations of the distal colon (Fig 12). The number of ChAT-IR neurons was similar in preparations from all groups at the 6 hour time point (sham: 156±2 cells/area, TNBS: 156±3 cells/area, MSC: 152±3 cells/area, CM: 153±2 cells/area, UCM: 146±2 cells/area, Figs 12A–12E and 13A). The number of ChAT-IR neurons decreased at the 24 and 72 hour time points in TNBS-only and UCM-treated groups (24 hours, TNBS: 108±2 cells/area, UCM: 115±2 cells/area; 72 hours, TNBS: 111±5 cells/area, UCM: 115±12 cells/area) compared to sham (24 hours: 158±7 cells/area, 72 hours: 155±2 cells/area), MSC (24 hours: 155±2 cells/area, 72 hours: 150±4 cells/area) and CM (24 hours: 147±3 cells/area, 72 hours: 150±4 cells/area) groups (*P<*0.001 for all). The number of ChAT-IR neurons was unaffected at 24 and 72 hours in MSC and CM-treated groups (Figs 12A^I^–12E^II^ and 13A). There were no differences between any groups in the proportion of ChAT-IR neurons to total number of neurons at all time points (Fig 13B).

### Neuroprotective factors released by MSCs

RT-PCR, flow cytometry and antibody array analysis of factors in conditioned medium revealed that MSCs used in our study released a substantial number of growth factors, cytokines and proteins exerting neuroprotective effects. Expression of factors with well-known neuroprotective functions have been detected by highly sensitive RT-PCR and flow cytometry techniques. The antibody array method was less sensitive than RT-PCR and flow cytometry, however the capacity to detect great amount of different target proteins enabled to identify many other neuroprotective factors released by MSCs (S3 Table).

## Discussion

The objective of this study was to determine the time point at which MSC-based therapies initiate their effects in the guinea-pig model of TNBS-induced colitis. The present study showed that MSC and CM treatments 1) prevented weight loss, 2) accelerated repair of colonic architecture, 3) reduced immune infiltrate both in the mucosa and at the level of the myenteric ganglia, 4) accelerated regeneration of nerve fibers and 5) attenuated the loss of myenteric neurons and changes in their subpopulations. Substantial number of neuroprotective factors released by MSCs has been detected in their secretome. Overall, prominent effects of MSC-based therapies were observed 24 hours after induction of inflammation in the guinea-pig model of TNBS-induced colitis.

We utilized human BM-MSCs since they are the best characterized and most established as a viable cellular therapy for many pathological conditions in both animal and clinical studies [58]. In our study, BM-MSCs were administered 3 hours after TNBS when the most significant damage to the mucosa occurs [57]. Timing of administration can impact MSC efficacy [59] and previous studies have shown that MSC transplantation at earlier phases of inflammation are optimal for therapeutic effects [60–62].

Viable human MSCs constitutively express MHC class I molecules on their surface at moderate levels [63] and can escape immune rejection when transplanted into guinea-pigs, as well as other species without the necessity of host immunosuppression [64]. It is known that chemokine receptors and adhesion molecules expressed by MSCs respond to chemoattractant signals from sites of injury facilitating MSC migratory and homing capacity [60]. We employed immuno-labelling of colon cross sections with anti-HLA-A,B,C antibody to evaluate the successful migration and engraftment of MSCs within the inflamed colon wall. Results of our study verified the migratory capacity of MSCs throughout the layers of the inflamed colon, but not to the uninflamed colon. Progressive MSC homing towards inflamed sites is evidenced by their presence in the colonic mucosa at 6 hours after induction of colitis and at the level of the myenteric plexus by 24 hours and 72 hours post inflammation.

The successful migration and engraftment of enema-applied MSCs into the inflamed colonic wall observed in our study is consistent with previous reports demonstrating implantation of locally administered MSCs into target tissues, especially in inflammatory conditions [44, 51]. However, enema application is less invasive than administration of MSCs into the injury site, such as injection directly into the colonic wall [44], and is safe, convenient and feasible for IBD patients without complications. Furthermore, enema application is a plausible administration route for CM treatments.

Weight loss associated with TNBS-induced colitis was observed in our study and is consistent with other reports [12, 20]. MSC-based therapies effectively attenuated weight loss at 24 hours post induction of colitis and at subsequent time points. Furthermore, administration of MSCs or CM into the distal colon ameliorated epithelial and goblet cell loss, as well as repaired colonic architecture by 24 hours after induction of colitis and was maintained for at least 72 hours. These findings are consistent with previous reports on the therapeutic efficiency of MSCs in other animal models of colitis where MSCs and CM have been shown to protect against ulceration, necrosis of epithelial tissue, and increased permeability of the mucosa [44, 45].

As we have shown in our model of experimental colitis, MSCs reduce leukocyte infiltrate which is also observed in other disease models [32, 45, 59]. The immunomodulatory mechanisms however, have not been fully elucidated, although it is considered to be through either the production of various soluble factors or by direct interaction with target cells [36, 65]. MSCs exhibit potent modulatory effects on immune cells including T and B lymphocytes, natural killer cells and dendritic cells [66, 67]. The ability of MSCs to exert their immunosuppressive function requires MSC activation in a pro-inflammatory microenvironment [68]. Our results indicated that MSC and CM treatments were effective within 24 hours post inflammation as confirmed by reduced leukocyte numbers throughout the distal colon wall. MSCs were able to continue inhibition of CD45-IR cells for 72 hours and can exert long lasting effects up to 7 days as shown in our previous study [18]. Infiltration of leukocytes to the level of myenteric ganglia was reduced in MSC and CM treated groups at 24 hours post induction of colitis, but was still higher than in the sham treated group. The level of CD45-IR cells was comparable to the sham group only by 72 hours.

Our study demonstrated a loss of myenteric neurons at 24 hours post induction of colitis. A reduction in the quantity of myenteric neurons following TNBS administration is consistent with previous studies in the guinea-pig intestine [10, 15]. Neuronal numbers were unaffected at 6 hours which is consistent with previous findings reporting no change in the total number of myenteric neurons 6 hours after TNBS administration [10]. MSC and CM treatments prevented the neuronal loss associated with TNBS-induced inflammation. The number of myenteric neurons in MSC and CM-treated groups was unchanged at all time points even though the quantity of leucocytes at the level of the myenteric ganglia remained increased at 24 hours. This indicates that MSC-based therapies are capable of exerting neuroprotective effects independent of anti-inflammatory actions. Some studies have reported the capacity of MSCs to differentiate into cells of neuronal and glial lineage [34, 35]. Despite evidence that MSCs have the ability to differentiate into neurons and glial cells after appropriate induction *in vitro*, it has been shown that MSCs transplanted into the damaged central nervous system infrequently differentiate into neural phenotypes [69, 70]. Limited cellular telomerase activity and a maximum 72 hour time point in the current study constrained the likelihood of observing any *in vivo* MSC differentiation into specific cell lineages [71–73].

In addition to preventing a reduction in the total number of myenteric neurons, MSCs accelerated regeneration and re-growth of nerve fibers in the colon. Damage to the nerve fibers was observed in all groups at 6 hours after induction of inflammation. However, in MSC and CM treated animals, re-growth of axons was evident by 24 hours and nerve fiber density returned to sham levels at 72 hours. Promotion of axonal re-growth by MSC-based therapies within this study is consistent with previous findings in models of spinal cord injury [74, 75].

Our study revealed an increase in the number of nNOS neurons, as well a decrease in the number of ChAT neurons at 24 hours after induction of colitis. These results are comparable to previous studies in TNBS and dextran sodium sulphate animal models of colitis, as well as in tissues from IBD patients reporting changes in neurochemical coding of enteric neuronal subpopulations [10, 16, 17, 76, 77]. Nitrergic (nNOS) neurons in the myenteric plexus are primarily inhibitory motor neurons responsible for the relaxation of intestinal smooth muscle, while cholinergic (ChAT) neurons are the major excitatory motor neurons of the ENS [7]. Imbalances in numbers of nNOS and ChAT neurons associate with impaired smooth muscle contractility and intestinal dysmotility [17]. We found that MSC-based therapies alleviated the inflammation-induced increase in nNOS neurons and loss of ChAT neurons in the myenteric plexus at 24 and 72 hours. These results are consistent with our previous study in which MSC-based therapies attenuated changes in subpopulations of nNOS and ChAT myenteric neurons and colon dysfunction 7 days post treatment [18].

We found that the efficacy of CM treatment was comparable to MSC therapy. Previous findings in nervous system injury and other pathological conditions highlight the therapeutic potential of soluble factors released by MSCs [36, 78–83]. It is now considered that the protective mechanisms and endogenous regeneration initiated by MSCs is due to their ability to produce and secrete a wide range of bioactive soluble factors acting as paracrine mediators; directly stimulating target cells and/or instigating nearby cells to release functionally active agents [84]. The MSC secretome contains anti-apoptotic, neurotrophic and growth factors, as well as neurotransmitters and can promote stimulation of axonal growth, microglial regulation, immunomodulation and re-myelination of host axons [85–87].

The MSC secretome may be responsible for the therapeutic effect observed in this study via: 1) immunomodulation by reducing *in vivo* levels of pro-inflammatory mediators while simultaneously increasing the production of anti-inflammatory factors in the gut and serum and/or 2) direct neuroprotection of enteric neurons via release of neurotrophic factors. During an inflammatory flare, an array of cytokines and chemokines, reactive oxygen and nitrogen species, prostaglandins and other pro-inflammatory mediators induce profound structural and functional damage to the ENS [88–91]. Previous studies in experimental colitis have demonstrated that MSC treatment stimulates upregulation of anti-inflammatory factors including interleukin (IL)-10, indoleamine 2,3-dioxygenase, prostaglandin E2, and transforming growth factor-β1, as well as a downregulation of pro-inflammatory mediators such as IL-1β, IL-6, IL-12, IL-17, interferon-γ and tumor necrosis factor-α [67, 92, 93]. This immunomodulatory effect of MSC therapy could in turn prevent and/or reduce the damage to enteric neurons and nerve fibers. A direct MSC effect for maintaining enteric neuronal integrity during intestinal inflammation may occur via release of neurotrophic and neuroprotective agents in the MSC secretome. In this study, analysis of conditioned medium revealed that MSCs released considerable number of factors under normal culturing conditions including those that have been demonstrated to be neurotrophic and neuroprotective for enteric neurons as well as factors that have not been studied in the ENS. Previous studies have shown that glial-derived neurotrophic factor protects enteric neurons from apoptosis and promotes the development and survival of many types of neurons [6, 94–97]. Other factors, such as neurotrophin, nerve growth factor, brain-derived neurotrophic factor, ciliary neurotrophic factor and leukemia inhibitory factor are also involved in modulation of gut inflammation and colonic sensitivity [94, 95]. In our study neurotrophic and neuroprotective factors were detected in the secretome of naïve MSCs (not exposed to inflammatory conditions). It is plausible that upon placement in an inflammatory milieu MSCs release a wider array of factors than we have reported in this study. The role of specific MSC-secreted neuroprotective factors in preventing enteric neuronal loss and damage caused by intestinal inflammation needs to be further elucidated.

In conclusion, this study confirmed that BM-MSCs and CM have the ability to prevent inflammatory insults to the ENS when administered 3 hours after induction of colitis. Our results clearly support the therapeutic potential of enema-administered MSC and CM treatments for preventing enteric neuropathy associated with TNBS-induced colitis as early as 24 hours after induction of inflammation even though the inflammatory reaction at the level of the myenteric ganglia has not completely subsided at this time point. This suggests that the neuroprotective efficacy of MSC-based therapies can be exerted independently to their anti-inflammatory effects. Further investigations need to be conducted to define the pathways and specific factors contributing to enteric neuroprotection by MSC-based treatments in acute and chronic inflammatory conditions.

## Supporting Information

S1 Dataset Body weight of guinea-pigs within 72 hours post induction of colitis or sham treatment. Guinea-pigs were weighed daily from day of treatment until up to 72 hours after treatment. Raw data of weight (g) at 24, 48 and 72 hours was calculated as a percentage of weight at day 0 (Sheet 1). These values were used to create Table 2 and Fig 2 (Sheet 2).
(XLSX)

S2 Dataset Gross morphological changes in the distal colon assessed in H&E stained cross sections. H&E stained cross sections of guinea-pig distal colon were assessed for gross morphological damage by histological grading of three parameters: mucosal flattening (0 = normal, 3 = severe flattening), occurrence of hemorrhagic sites (0 = none, 3 = frequent sites), and variation of the circular muscle (0 = normal, 3 = considerable thickening of muscular layer). Scores were averaged per group per time point (Sheet 1).
(XLSX)

S3 Dataset MSC and CM treatments attenuated the immune response in the distal colon 24 hours after induction of inflammation-Cross sections. CD45-IR was measured in cross sections of the distal colon. Cells were counted within 8 images per animal. The sum of CD-IR cells per guinea-pig was averaged per group.
(XLSX)

S4 Dataset MSC and CM treatments attenuated the immune response in the distal colon 24 hours after induction of inflammation. CD45-IR was assessed in LMMPs of the distal colon. Cells were counted within 8 images per animal. The sum of CD45-IR cells per guinea-pig was averaged per group.
(XLSX)

S5 Dataset MSC and CM treatments facilitated re-growth of nerve fibers 24 hours after induction of colitis. Nerve fibers in cross sections of the distal colon were labelled by neuron specific anti-β-tubulin (III) antibody. Image J was used to assess the density of fibers in all groups at all time points and presented as a percent area of β-tubulin (III)-IR.
(XLSX)

S6 Dataset MSC and CM treatments protected against neuronal loss 24 hours after induction of colitis. The total number of Hu-IR neurons was counted in 8 images from each animal per group per time point. The sum of Hu-IR neurons counted in LMMPs from each animal was averaged per group and time point and presented as a mean.
(XLSX)

S7 Dataset Effects of MSC and CM treatments on inhibitory neurons in the distal colon. The total number of nNOS-IR neurons was counted in 8 images from each animal per group per time point. The sum of nNOS-IR neurons counted in LMMPs from each animal was averaged per group and time point and presented as a mean. The proportion of nNOS-IR neurons to the total number of neurons was calculated by dividing the mean sum of nNOS-IR neurons per group per time point with the total number of neurons x 100.
(XLSX)

S8 Dataset Effects of MSC and CM treatments on excitatory neurons in the distal colon. The total number of ChAT-IR neurons was counted in 8 images from each animal per group per time point. The sum of ChAT-IR neurons counted in LMMPs from each animal was averaged per group and time point and presented as a mean. The proportion of ChAT-IR neurons to the total number of neurons was calculated by dividing the mean sum of ChAT-IR neurons per group per time point with the total number of neurons x 100.
(XLSX)

S1 Table Primers for RT-PCR. (DOC)

S2 Table Body weight of guinea-pigs within 72 hours post induction of colitis or sham treatment. (DOC)

S3 Table Neuroprotective factors released by MSCs used in our study. (DOC)
