# Supplementary material for: Neuroprotective Potential of Mesenchymal Stem Cell-Based Therapy in Acute Stages of TNBS-Induced Colitis in Guinea-Pigs
Source: PLoS One. 2015 Sep 23;10(9):e0139023. doi: 10.1371/journal.pone.0139023 (PMC4580595; doi:10.1371/journal.pone.0139023)
Supplement: S1 Table — (DOC) [file pone.0139023.s009.doc]

**S1 Table.** Primers for RT-PCR

| **Target** | **Forward** | **Reverse** | **Product size** |
| --- | --- | --- | --- |
| β-actin | CAGAGCCTCGCCTTTGCCG | CTCGCGGTTGGCCTTGGG | 405 |
| BDNF | AGAGGCTTGACATCATTGGC | ACTAATACTGTCACACACGC | 276 |
| NGF | CACACTGAGGTGCATAGCGT | TGATGACCGCTTGCTCCTGT | 390 |
| IGF-1 | ATG CAC ACC ATG TCC TC | CATCCTGTAGTTCTTGTTTC | 390 |
| NT3 | ATCTTACAGGTGAACAAGGT | TCGGTGACTCTTATGCTCCG | 459 |
| HGF | ATGCATCCAAGGTCAAGGAG | TTCCATGTTCTTGTCCCACA | 349 |
| VEGF | ATGAACTTTCTGCTGTCTTGG | TCACCGCCTCGGCTTGTCACA | 516 |
| TSG-6 | AAG CAC GGT CTG GCA AAT ACA AGC | GGGTTGTAGCAATAGGCATCC | 268 |
| TGF β1 | CAGATCCTGTCCAAGCTG | TCGGAGCTCTGATGTGTT | 270 |
