# Supplementary material for: Neuroprotective Potential of Mesenchymal Stem Cell-Based Therapy in Acute Stages of TNBS-Induced Colitis in Guinea-Pigs
Source: PLoS One. 2015 Sep 23;10(9):e0139023. doi: 10.1371/journal.pone.0139023 (PMC4580595; doi:10.1371/journal.pone.0139023)
Supplement: S2 Table — (DOC) [file pone.0139023.s010.doc]

**S2 Table.**

****Body weight of guinea-pigs**** within 72 hours post induction of colitis or sham treatment

| **Time points**  **Groups** | **0 hours** | **6 hours** | **24 hours** | **48 hours** | **72 hours** |
| --- | --- | --- | --- | --- | --- |
| **Sham** | 100.0 | 100.9 ± 0.2 | 102.4 ± 1.0 | 105.2 ± 0.9 | 107.2 ± 0.5 |
| **TNBS** | 100.0 | 100.2 ± 0.2 | 95.4 ± 1.4* | 97.9 ± 0.6* | 97.2 ± 2.4* |
| **TNBS+MSC** | 100.0 | 100.3 ± 0.3 | 102.7 ± 1.1 | 104.5 ± 1.4 | 106.3 ± 1.6 |
| **TNBS+CM** | 100.0 | 100.0 ± 0.1 | 100.9 ± 0.3 | 103.1 ± 1.0 | 107.5 ± 1.4 |
| **TNBS+UCM** | 100.0 | 100.0 ± 0.1 | 98.8 ± 0.7^ | 99.3 ± 1.7* | 100.2 ± 2.1* |

******P*<.05 significantly different to sham, TNBS+MSC and TNBS+CM groups.

**^***P*<.05 significantly different to sham and TNBS+MSC groups.
