# Supplementary material for: Neuroprotective Potential of Mesenchymal Stem Cell-Based Therapy in Acute Stages of TNBS-Induced Colitis in Guinea-Pigs
Source: PLoS One. 2015 Sep 23;10(9):e0139023. doi: 10.1371/journal.pone.0139023 (PMC4580595; doi:10.1371/journal.pone.0139023)
Supplement: S3 Table — (DOC) [file pone.0139023.s011.doc]

**S3 Table**

Neuroprotective factors released by MSCs used in our study

| **Factors detected in MSC secretome in our study** | | **Neuroprotective role established in published studies** |
| --- | --- | --- |
| **Factor** | **Detection method** |
| NGF | RT-PCR | Neuroprotective for hypoxic-ischemic injuries of the brain, optic pathways, and skin, rescues extraocular motoneurons from axotomy-induced cell death [98, 99] |
| NT3 | RT-PCR | Neuroprotective against focal cerebral ischemia/reperfusion injury in rats, rescues extraocular motoneurons from axotomy-induced cell death [99, 100] |
| BDNF | RT-PCR Antibody array | Amplifies neurotransmitter responses and promotes synaptic communication in the ENS, rescues extraocular motoneurons from axotomy-induced cell death [99, 101] |
| TSG-6 | RT-PCR | Improves neurological function after global cerebral ischemia [102] |
| VEGF | RT-PCR | Protective factor in hypoxia–ischemia, in vitro excitotoxicity, motor neuron degeneration and against seizure-induced neuronal loss in hippocampus [103] |
| GDNF | RT-PCR | Rescues extraocular motoneurons from axotomy-induced cell death, protects enteric glia from apoptosis [97, 99] |
| HGF | RT-PCR | Morphologic and physiological preservation of photoreceptors in rats with photoreceptor degeneration induced by phototoxicity or a gene mutation [104] |
| IL-6 | Flow cytometry | Neuroprotective effect in brain ischemic injury [105] |
| TGF beta 1 TGF beta 3 | RT-PCR Antibody array  Flow cytometry | Neuroprotective function following brain ischemia, trauma, sclerosis multiplex, neurodegenerative diseases, infections, and brain tumours [106] |
| IGF1 | RT-PCR | Protects neurons against cell death induced by amyloidogenic derivatives, glucose or serum deprivation relating Alzheimer's disease and other neurodegenerative disorders [107] |
| BMP-4 | Antibody array | Protects retinal neurons [108] |
| Cardiotrophin-1 /CT1 | Antibody array | Protects motor, sensory and sympathetic neurons in the peripheral nervous system [109] |
| Frizzled 5 | Antibody array | Neuroprotection for nervous system development [110] |
| GPX3 | Antibody array | Neuroprotective in Huntington's disease [111] |
| Activin B | Antibody array | Neuroprotective activity associated with regulation of Bcl-2 family proteins [112] |
| Axl | Antibody array | Alleviates progression of experimental autoimmune encephalomyelitis, an animal model for multiple sclerosis [113] |
| PAI-1 | Antibody array | Anti-apoptotic role in CNS neurons [114] |
| IFN-beta | Antibody array | Reduces disease burden in relapsing-remitting multiple sclerosis patients [115] |
| CCR1 | Antibody array | May be associated with specific activities of human MSCs after their migration to the target lesions [116] |
| Chordin-Like 1 | Antibody array | Promotes neuronal differentiation [117] |
| Fibronectin | Antibody array | Promotes neuron-glial extrasynaptic transmission in Parkinson's disease [118] |
| Frizzled 1 | Antibody array | Neuroprotective for dopaminergic neurons and astrocytes [119] |
| Glypican 5 | Antibody array | Neuroprotective for neuronal development and axon guidance; expression in adult brain tissue suggests a possible role in controlling neurotropic factors and maintaining neural function [120] |
| GREMLIN | Antibody array | Protects substantia nigra dopamine neurons and several dopamine cell lines in MPTP mouse model of Parkinson's disease [121] |
| IL-1 alpha | Antibody array | Neuroprotective effect against an excitotoxic challenges with NMDA [122] |
| MIP-1a | Antibody array | Prevents neuronal cell death associated with gp120 [123] |
| SPARC | Antibody array | Retinal ganglion cell neuroprotection [124] |
| TIMP-1 | Antibody array | Neuroprotective against traumatic and ischemic brain injury in mice [125] |

**References**

1. Chiaretti A, Falsini B, Aloe L, Pierri F, Fantacci C, Riccardi R. Neuroprotective role of nerve growth factor in hypoxic-ischemic injury. From brain to skin. Archives Italiennes de Biologie. 2011;149: 275-82.
2. Morcuende S, Munoz-Hernandez R, Benitez-Temino A, Pastor AM, de la Cruz RR. Neuroprotective effects of ngf, bdnf, nt-3 and gdnf on axotomized extraocular motoneurons in neonatal rats. Neurosci. 2013;250: 31-48.
3. Zhang J, Shi Q, Yang P, Xu X, Chen X, Qi C, Zhang J, et al. Neuroprotection of neurotrophin-3 against focal cerebral ischemia/reperfusion injury is regulated by hypoxia-responsive element in rats. Neurosci. 2012;222: 1-9.
4. Boesmans W, Gomes P, Janssens J, Tack J, Vanden Berghe P. Brain-derived neurotrophic factor amplifies neurotransmitter responses and promotes synaptic communication in the enteric nervous system. Gut. 2008;57: 314-22.
5. Lin Q, Zhao S, Zhou L, Fang XS, Fu Y, Huang ZT. Mesenchymal stem cells transplantation suppresses inflammatory responses in global cerebral ischemia: contribution of TNF-α-induced protein 6. Acta Pharmacol Sin. 2013;34: 784-92.
6. Nicoletti JN, Shah SK, McCloskey DP, Goodman JH, Elkady A, Atassi H, et al. Vascular endothelial growth factor is upregulated after status epilepticus and protects against seizure-induced neuronal loss in hippocampus. Neurosci. 2008;151: 232-41.
7. Machida S, Tanaka M, Ishii T, Ohtaka K, Takahashi T, Tazawa Y. Neuroprotective effect of hepatocyte growth factor against photoreceptor degeneration in rats. IOVS. 2004;45: 4174-82.
8. Jung JE, Kim GS, Chan PH. Neuroprotection by IL-6 Is mediated by STAT3 and antioxidative signaling in ischemic stroke. Stroke. 2011;42: 3574-79.
9. Dobolyi A, Vincze C, Pal G, Lovas G. The neuroprotective functions of transforming growth factor beta proteins. Int J Mol Sci. 2012;13: 8219-58.
10. Zheng W, Kar S, Dore S, Quirion R. Insulin-like growth factor-1 (IGF-1): a neuroprotective trophic factor acting via the Akt kinase pathway. J Neural Transm Suppl. 2000;60: 261-72.
11. Fischer AJ, Schmidt M, Omar G, Reh TA. BMP4 and CNTF are neuroprotective and suppress damage-induced proliferation of Müller glia in the retina. Mol Cell Neurosci. 2004;27: 531-42.
12. Sola A, Peng H, Rogido M, Wen TC. Animal models of neonatal stroke and response to erythropoietin and cardiotrophin-1. Int J Dev Neurosci. 2008;26: 27-35.
13. Slater PG, Ramirez VT, Gonzalez-Billault C, Varela-Nallar L, Inestrosa NC.Frizzled-5 receptor is involved in neuronal polarity and morphogenesis of hippocampal neurons. PLoS One. 2013;8: e78892.
14. Mason RP, Casu M, Butler N, Breda C, Campesan S, Clapp J, et al. Glutathione peroxidase activity is neuroprotective in models of Huntington’s disease. Nat Genet. 2014;45: 1249-54.
15. Kupershmidt L, Amit T, Bar-Am O, Youdim MB, Blumenfeld Z, et al. The neuroprotective effect of Activin A and B: implication for neurodegenerative diseases. J Neurochem. 2007;103: 962-71.
16. Weinger JG, Brosman CF, Loudig O, Goldberg MF, Macian F, Arnett HA, et al. Loss of the receptor tyrosine kinase Axl leads to enhanced inflammation in the CNS and delayed removal of myelin debris during experimental autoimmune encephalomyelitis. J Neuroinflamm. 2011;8: 49.
17. Soeda S, Koyanagi S, Kuramoto Y, Kimura M, Oda M, Kozako T, et al. Anti-apoptotic roles of plasminogen activator inhibitor-1 as a neurotrophic factor in the central nervous system. Thromb Haemost. 2008;100: 1014-20.
18. Croze E, Yamaguchi KD, Knappertz V, Reder AT, Salamon H.. Interferon-beta-1b-induced short- and long-term signatures of treatment activity in multiple sclerosis. Pharmacogenomics J. 2013;13: 443-51.
19. Song CH, Honmou O, Furuoka H, Horiuchi M. Identification of chemoattractive factors involved in the migration of bone marrow-derived mesenchymal stem cells to brain lesions caused by prions. J Virol. 2011;85: 11069-78.
20. Gaughwin P, Ciesla M, Yang H, Lim B, Brundin P.Stage-specific modulation of cortical neuronal development by Mmu-miR-134. Cereb Cortex. 2011;21:1857-69.
21. Wang J, Yin L, Chen Z. Neuroprotective role of fibronectin in neuron-glial extrasynaptic transmission. Neural Regen Res. 2013;8: 376-82.
22. Episcopo FL, Serapide MF, Tirolo C, Testa N, Caniglia S, Morale MC, et al. A Wnt1 regulated Frizzled-1/b-Catenin signaling pathway as a candidate regulatory circuit controlling mesencephalic dopaminergic neuron astrocyte crosstalk: therapeutical relevance for neuron survival and neuroprotection. Mol Neurodegen. 2011;6: 49.
23. Thway K, Selfe J, Shipley J. GPC5 (glypican 5). Appl Immunohistochem Mol Morphol. 2012;20: 189-95.
24. Phani S, Jablonski M, Pelta-Heller J, Cai J, Iacovitti L. Gremlin is a novel VTA derived neuroprotective factor for dopamine neurons. Brain Res. 2013;1500:88-98.
25. Rogers SW, Carlson NG, Weiggel WA, Chen J. Inflammatory cytokines IL-1a, IL-1b, IL-6, and TNF- a impart neuroprotection to an excitotoxin through distinct pathways. J Immunol. 1999;163: 3963-68.
26. Brenneman DE, Hauser J, Spong CY, Phillips TM. Chemokines released from astroglia by vasoactive intestinal peptide. Mechanism of neuroprotection from HIV envelope protein toxicity. Ann N Y Acad Sci. 2000; 921:109-14.
27. Johnson TV, DeKorver NW, Levassuer VA, Osborne A, Tassoni A, Lorber B, et al. Identification of retinal ganglion cell neuroprotection conferred by platelet-derived growth factor through analysis of the mesenchymal stem cell secretome. Brain. 2014;137: 503-19.
28. Tejima E, Guo S, Murata Y, Arai K, Lok J, van Leyen K, et al. Neuroprotective effects of overexpressing tissue inhibitor of metalloproteinase TIMP-1. J Neurotrauma. 2009;26: 1935-41.
